# Evaluating the accessibility and value of U.S. ambulatory care among Medicaid expansion states and non-expansion states, 2012–2015

**DOI:** 10.1186/s12913-023-09696-x

**Published:** 2023-07-03

**Authors:** Aaron Parzuchowski, Carlos Oronce, Rong Guo, Chi-Hong Tseng, A. Mark Fendrick, John N. Mafi

**Affiliations:** 1 Department of Veteran Affairs, National Clinician Scholars Program, Ann Arbor, MI USA; 2 Division of General Medicine, University of Michigan, Ann Arbor, MI USA; 3 Division of General Internal Medicine and Health Services Research, University of California Los Angeles, Los Angeles, CA USA; 4 VA Greater Los Angeles Healthcare System, Los Angeles, CA USA

**Keywords:** Quality, Value, Medicaid, Ambulatory care, Health policy, Affordable care act

## Abstract

**Background:**

While the Affordable Care Act’s Medicaid expansion improved healthcare coverage and access for millions of uninsured Americans, less is known about its effects on the overall accessibility and quality of care across all payers. Rapid volume increases of newly enrolled Medicaid patients might have unintentionally strained accessibility or quality of care. We assessed changes in physician office visits and high- and low-value care associated with Medicaid expansion across all payers.

**Methods:**

Prespecified, quasi-experimental, difference-in-differences analysis pre and post Medicaid expansion (2012–2015) in 8 states that did and 5 that did not choose to expand Medicaid. Physician office visits sampled from the National Ambulatory Medical Care Survey, standardized with U.S. Census population estimates. Outcomes included visit rates per state population and rates of high or low-value service composites of 10 high-value measures and 7 low-value care measures respectively, stratified by year and insurance.

**Results:**

We identified approximately 143 million adults utilizing 1.9 billion visits (mean age 56; 60% female) during 2012–2015. Medicaid visits increased in expansion states post-expansion compared to non-expansion states by 16.2 per 100 adults (*p* = 0.031 95% CI 1.5–31.0). New Medicaid visits increased by 3.1 per 100 adults (95% CI 0.9–5.3, *p* = 0.007). No changes were observed in Medicare or commercially-insured visit rates. High or low-value care did not change for any insurance type, except high-value care during new Medicaid visits, which increased by 4.3 services per 100 adults (95% CI 1.1–7.5, *p* = 0.009).

**Conclusions:**

Following Medicaid expansion, the U.S. healthcare system increased access to care and use of high-value services for millions of Medicaid enrollees, without observable reductions in access or quality for those enrolled in other insurance types. Provision of low-value care continued at similar rates post-expansion, informing future federal policies designed to improve the value of care.

**Supplementary Information:**

The online version contains supplementary material available at 10.1186/s12913-023-09696-x.

## Background

Following a U.S. Supreme Court ruling in 2012, the Affordable Care Act’s (ACA) Medicaid expansion became optional. This ruling led to state-level variation in enrollment increases among states that opted to expand Medicaid eligibility (effective January 1, 2014). Twenty-eight states and the District of Columbia chose to expand Medicaid in 2014. Within two years, the number of adults without health insurance declined by 17.7 million [1]; a decline that was significantly larger in states that expanded Medicaid than in states that did not [2]. Additionally, the percentage of Medicaid patients in primary care physicians’ panels increased in expansion states following expansion, but not in non-expansion states [3].

Many studies have assessed the effects of the ACA and Medicaid expansion in different settings such as emergency visits [4–6] or on specific preventative services [7–9] or access to ambulatory care [10–15] largely observing a benefit for uninsured patients with some positive effects on quality of care. Nevertheless, while Medicaid expansion’s effects on healthcare coverage and access for uninsured Americans is well documented, less is known about its effects on the overall accessibility and quality of care across the broader U.S. healthcare system. This is particularly important in the context of the recent *Braidwood Management v Becerra* ruling, challenging coverage of preventive services without cost-sharing under ACA Sect. 2713 [16]. There could be decreased utilization of high-value preventive services with cost-sharing imposed on patients.

Because the ACA did not directly address physician supply, the rapid increase in Medicaid enrollment and demand for care raised concerns that expansion would have negative unintended consequences [17–19]. First, might new patients crowd out existing patients with Medicaid and other insurance, resulting in prolonged or delayed appointment times, reduced clinician availability, and therefore, unintentionally compromise access to care for patients with Medicaid and other insurance? This concern is particularly salient in the context of increasingly narrow physician networks within Medicaid and commercial exchange plans [20]. Second, increased clinical workload of integrating new patients into full patient panels, especially among the limited number of clinicians willing to accept Medicaid patients, could lead to erosions in quality of care that would affect both new patients and existing ones. To deal with the rapid rise in patient volume, for example, might some physicians feel pressured to deliver more inefficient, low-value care? Some analyses have reported no negative spillover to the Medicare population [21] and negative spillover to the commercial population [22], but neither assessed changes in spillover of the value of care. To address this evidence gap, we sought to assess the effects of Medicaid expansion on the overall accessibility and quality of care for Medicaid, Medicare, and commercially insured patients.

## Methods

We hypothesized that Medicaid expansion would be associated with increases in Medicaid visits without decreases in visits for other insurance types; as more individuals were enrolled in Medicaid, the demand, and subsequent utilization for healthcare services would increase. Additionally, Medicaid visits in expansion states would be associated with increases in both high- and low-value care. To test these hypotheses, we utilized visit-level data from the National Ambulatory Medical Care Survey (NAMCS), standardized with state-level U.S. Census population estimates. We used prespecified difference-in-differences (DinD) analytical approaches to assess changes in physician office visits and high- and low-value care use between expansion states and non-expansion states before and after Medicaid expansion across all-payers.

### Data source and collection

NAMCS is a nationally representative cross-sectional survey of visits to office-based outpatient practices. The National Center for Health Statistics (NCHS) oversees NAMCS using a complex, multistage probability design detailed elsewhere [23]. For sampled visits, NAMCS collects information from the medical record, including patient demographics, payer, reasons for the visit, diagnoses, comorbidities, procedures, diagnostic tests, and medications. NAMCS includes survey weights that allow for national and regional estimates as well as state-level estimates during 2012–2015. We did not include the separate NAMCS community health center (CHC) sampling files in this analysis, which samples Federally Qualified Health Centers.

A DinD analysis using only NAMCS would not be able to account for changes in the underlying state population size as a potential confounder associated with changes in access to care unrelated to Medicaid expansion among expansion and non-expansion states. Therefore, we used U.S. Census Bureau state-level population estimates for 2012–2015 to account for time-varying population changes.

### Study sample

NAMCS provides state-level estimates for only years 2012–2015 and only for the most populous U.S. states (34 states in 2012, 22 in 2013, 18 in 2014, 16 in 2015). We included adult (age > 17) visits from 2012 to 2015 occurring in states (13) with state-level estimates available for all study years (expansion states: AZ, CA, IL, MA, NJ, NY, OH and WA; non-expansion states: FL, GA, NC, TX, and VA). We excluded non-adult visits and those occurring in states without state-level estimates. In addition to these adult visits, we identified subpopulations of visits by payer (Medicaid, Medicare, and commercially insured – defined as charges paid in-part or in-full by a private insurer (e.g., Blue Cross/Blue Shield) either directly to the physician or reimbursed to the patient; includes charges covered under a private insurance sponsored prepaid plan), and an additional subpopulation of “new” Medicaid patients. As NAMCS does not allow for individual identification of patients who enrolled in Medicaid as newly eligible under Medicaid expansion, we defined a new Medicaid visit as an adult visit with insurance type Medicaid, with a patient who has not been seen in that clinic before, to a clinician accepting new Medicaid patients.

Because Medicaid expansion was limited to the low-income adult population, we expected that this would be less likely to differentially affect visit rates or visit-level quality for older adults in the Medicare population (except the small number of dually-eligible enrollees in our sample) or for the commercially-insured population in expansion vs. non-expansion states. Therefore, these groups could theoretically serve as an alternative within expansion-state control group, in addition to the non-expansion states when considering results in the Medicaid population.

### Exposure - expansion status

The primary exposure of interest was whether a state expanded Medicaid as of January 1, 2014, identified using the Kaiser Family Foundation Medicaid Expansion Tracker [24]. Our treatment group included 8 expansion states, with 5 non-expansion states in the control group (listed above). We defined the pre-expansion period to include visits in years 2012 and 2013 and post-expansion for visits in 2014 and 2015.

### Primary accessibility and quality of care outcome measures

Our analysis had two categories of outcomes: access to care measured by physician office visit volume and quality of care measured by widely accepted high- and low-value care metrics. To measure visit volume, we counted all physician office visits in a survey year and summarized for each insurance type of interest (Medicaid, Medicare, and commercial). To assess quality of care, we used two composite outcome measures defined as receipt of any high-value care and receipt of any low-value care. (We pre-specified a composite given concern about sample size for individual measures within each subpopulation). We selected these individual measures based on prior literature and studies using NAMCS (including our own work) and professional physician practice guidelines, such as those developed by the USPSTF [25], National Committee for Quality Assurance [26], or Choosing Wisely^[Ⓡ]^[27]. (See Appendix). [28–41]. The high-value care outcome included ten measures: prescriptions for antiplatelet, statins, and beta blockers in coronary artery disease; beta blockers, angiotensin-converting enzyme inhibitor (ACEi) or angiotensin receptor blockers (ARB) in heart failure, anticoagulants in atrial fibrillation, antiplatelet agents in cerebrovascular disease, statins in diabetes mellitus, treatment for depression, and treatment for osteoporosis. The low-value care outcome included seven measures: screening for asymptomatic bacteriuria, screening for cardiovascular disease in low-risk patients, antibiotics for upper respiratory tract infections (URIs), opioid prescriptions for headache, opioid prescriptions for neck/back pain, advanced imaging for headache, and advanced imaging for neck/back pain. For each measure comprising the outcome, we followed previously established inclusion and exclusion criteria relying on presence of reason for visit codes, diagnostic codes, and comorbidity indicators to identify eligible visits. We determined whether a patient received any of the above high or low-value services in a similar way using prescription drug codes and whether the clinician ordered the related diagnostic imaging or lab.

### Statistical analysis

To mitigate publication and/or selective reporting bias, the study protocol was pre-registered on clinicaltrials.gov (NCT05319743). We first compared visit-level characteristics between Medicaid expansion and non-expansion states in the pre-expansion and post-expansion periods using descriptive statistics. We organized the subsequent analysis into three parts, in which the units of analysis for the first and third parts are state-year and the units of analysis for the second part are individual visits. All analyses use cluster-robust standard errors at the state-level to account for state-level clustering and sample weights to account for the complex survey design and non-response bias in accordance with NCHS guidelines [23].

First, we assessed differences in access to care by quantifying the number of Medicaid visits at the state-level, standardizing for state population. As such, we analyzed and reported visit changes as the number of visits per 100 adults. We then calculated survey-weighted Medicaid visit rates and parameters for expansion states and non-expansion states for each year. We then performed a DinD linear combination calculation of the rates and parameters to assess for significant changes pre and post expansion between expansion states and non-expansion states.

Second, we examined differences in the visit-level receipt of high or low-value care using multivariable logistic regression models, accounting for non-linearity of the logit-model. The model included binary indicators for state expansion status, pre- versus post-expansion period, the interaction of the expansion status and timeframe indicators, and visit-level adjustment variables. This model adjusted for visit-level characteristics that may confound the relationship between Medicaid expansion and quality measures including patient age, sex, race and/or ethnicity, rural versus urban location, and number of chronic conditions. To facilitate interpretability of the regression results, we reported average marginal effects (predicted probabilities). To account for the possibility that differential changes in diagnoses associated with visits over time could bias quality of care in expansion versus non-expansion states, a visit was only included in this analysis if it had the potential to result in a high- or low-value care. For example, visits for heart failure were included given the potential to prescribe several high-quality drugs; visits for back pain were included but excluded if there was a diagnosis of osteomyelitis requiring MRI; whereas visits for hand laceration were excluded as there is no corresponding high or low-value service to be performed in that visit.

Third, we examined differences in state-level rates of low-value and high-value care. We calculated our outcomes of interest for each state-year as a rate per 100 adults, summarizing the number of visits in which high and low-value care was provided, divided by the state adult population, and multiplied by 100. We again generated survey-weighted, visit rates and parameters for expansion states and non-expansion states for each year, followed by a DinD linear combination calculation of the rates and parameters to assess for significant changes pre- and post-expansion between expansion states and non-expansion states.

We repeated the above analyses substituting the Medicaid visits with all adult visits (Medicaid, Medicare, and commercially insured), Medicare visits, and commercially insured visits.

The DinD design has been used in multiple evaluations of Medicaid expansion and a key assumption for DinD analysis is that the time trend in the outcome variables during the pre-policy implementation period are parallel [42]. We successfully verified parallel trends using visual plots and formal placebo testing of the assumption by restricting the sample to the pre-expansion period and interacting expansion status with a linear term for year during 2012 versus 2013, which was non-significant.

We also accounted for multiple testing by applying the Benjamini-Hochberg step up procedure with a false discovery rate at the 5% level, [43] a level previously designated in published analyses [44]. We applied this correction to Medicaid and new Medicaid outcomes given multiple testing in these populations as our primary population of interest. We reported the p-values and whether they maintain significance following the multiple testing correction. We performed all analyses in Stata SE, version 17.0 (StataCorp, TX).

## Results

### Demographics

We identified approximately 1.9 billion adult physician office visits during 2012–2015; mean age 56 years, 60.0% female, 66.4% non-Hispanic white (Table 1). Approximately 0.7 billion visits occurred in non-expansion states (56.9 million adults) and 1.2 billion visits (62%) occurred in expansion states (86.2 million adults).

**Table 1:** Characteristics of study sample (NAMCS Adult Visits 2012–2015)

Characteristic	Non-Expansion		Expansion		*P* Value*
States (#)	5		8		
State Population	56,883,413		86,168,864		
	Pre-Expansion	Post-Expansion	Pre-Expansion	Post-Expansion	
Observations (Unweighted Adult Visits)	16,304	11,370	24,210	19,315	
Weighted Adult Visits	363,304,730	365,396,561	555,332,020	610,880,554	
Mean (SD) age (yr)	56.1 (14.9)	55.9 (12.2)	54.2 (14.5)	56.8 (12.2)	0.047
Female Sex (%)	61	62	58	59	0.916
Metropolitan Statistical Area (%)	93	95	97	97	0.302
Chronic Conditions (%)					0.242
0	33	29	37	29	
1	27	27	26	27	
2+	40	44	36	44	
Race/Ethnicity (%)					0.302
Non-Hispanic White	68	65	70	63	
Non-Hispanic Black	12	14	7	9	
Hispanic	17	17	16	16	
Non-Hispanic Other	3	4	7	12	

*The *p*-value of the interaction term of a DinD comparison

### Part 1: visit rate changes (state population level)

**Fig. 1:**
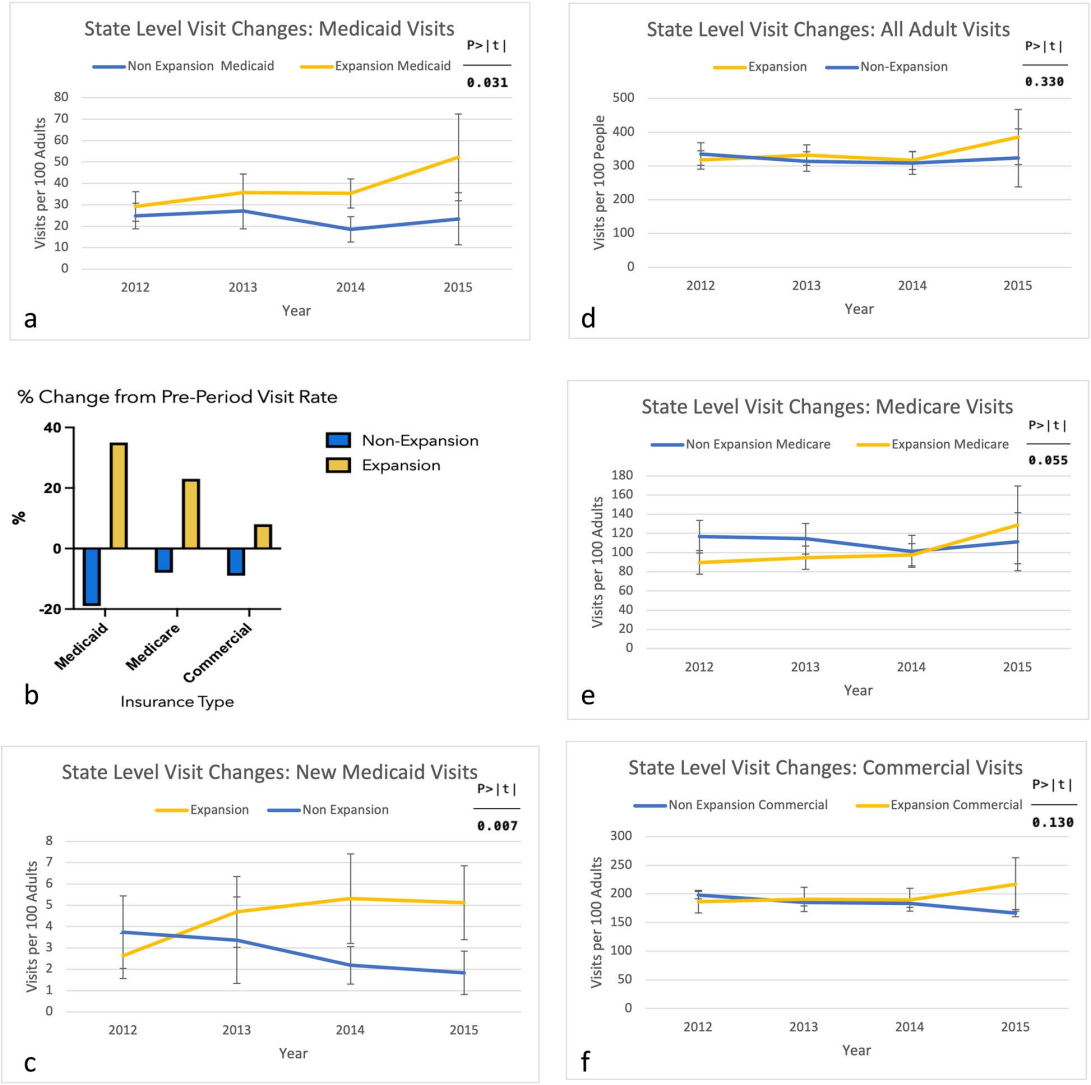
State-level changes in visit rates 2012–2015 for Medicaid visits (**a**), New Medicaid visits (**c**), all adult visits (**d**), Medicare visits (**e**), Commercial visits (**f**) and % change in rate in post period compared to pre period for Medicaid, Medicare, and Commercial visits (**b**)

We first assessed for changes in physician office visit rates between expansion states and non-expansion states post-expansion, standardized for state populations. For our primary population of Medicaid visits (Fig. 1), rates significantly increased post-expansion in expansion states compared to non-expansion states by 16.2 visits per 100 adults (*p* = 0.031 95% CI 1.5–31.0, significance maintained with correction). New Medicaid visits significantly increase in expansion states post-expansion by 3.1 visits (*p* = 0.007 95% CI 0.9–5.3, significance maintained with correction).

No significant change was identified among all adult visits, Medicare visits or commercially-insured visits. (Fig. 1). Though there was no significant increase in all adult visits, Medicaid visits increased without a substitution for other insurance types.

### Part 2: low-value and high-value care probabilities (visit level)

We then tested the adjusted visit-level probability of high-value or low-value care between expansion states and non-expansion states post-expansion (Table 2). New Medicaid visits did have significant increases in the predicted probability of receiving high-value care in expansion states (19% increase) post-expansion compared to non-expansion states (24% decrease), and significance remained after multiple-testing correction. There was no change in high- or low-value care for all Medicaid visits between expansion states and non-expansion states, post-expansion. Low-value care for the new Medicaid population could not be analyzed secondary to low sample size to reliably report this outcome (< 30 observations/cell per NCHS guidelines) [20]. Additionally, all adult, Medicare, and commercially-insured visits did not demonstrate significant changes in quality of care.

**Table 2:** Visit-level adjusted probability of low or high value service

		Non-Expansion		Expansion		
		Pre	Post	Pre	Post	
**Population**		% (95% CI)	% (95% CI)	% (95% CI)	% (95% CI)	***P***-Value*
Low Value	All Adults	39 (34–44)	45 (39–51)	33 (29–37)	39 (33–46)	0.893
	Medicaid	43 (31–56)	45 (24–66)	43 (35–52)	41 (33–48)	0.724
	Medicare	49 (40–58)	53 (44–63)	53 (44–61)	57 (48–65)	0.973
	Commercial	37 (32–43)	50 (42–58)	29 (25–33)	36 (28–44)	0.521
High Value	All Adults	41 (36–47)	46 (39–52)	43 (38–48)	51 (44–57)	0.526
	Medicaid	47 (33–61)	45 (30–60)	50 (39–62)	51 (39–63)	0.777
	Medicare	44 (35–52)	53 (43–63)	45 (37–54)	53 (43–62)	0.804
	Commercial	40 (34–45)	44 (26–51)	41 (35–47)	51 (43–47)	0.307
	New Medicaid	41 (18–64)	17 (-4–39)	46 (24–69)	65 (50–81)	**0.012**

*P value for the logistic regression interaction term between expansion status (expansion state/non-expansion state) and time period (pre/post expansion). Adjusted for age, sex, number of chronic conditions, race/ethnicity, metropolitan statistical area

### Part 3: low value and high value care rate changes (State Population Level)

For part three, we assessed if rates of low- or high-value care per 100 adults differed between expansion states and non-expansion states, post-expansion.

**Fig. 2:**
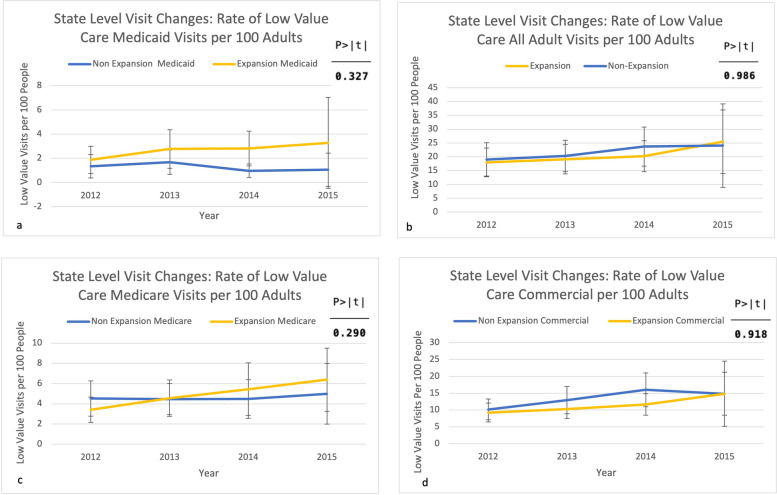
State-level low-value visit rate changes for Medicaid (**a**), all adult visits (**b**), Medicare (**c**), and Commercial (**d**)

**Fig. 3:**
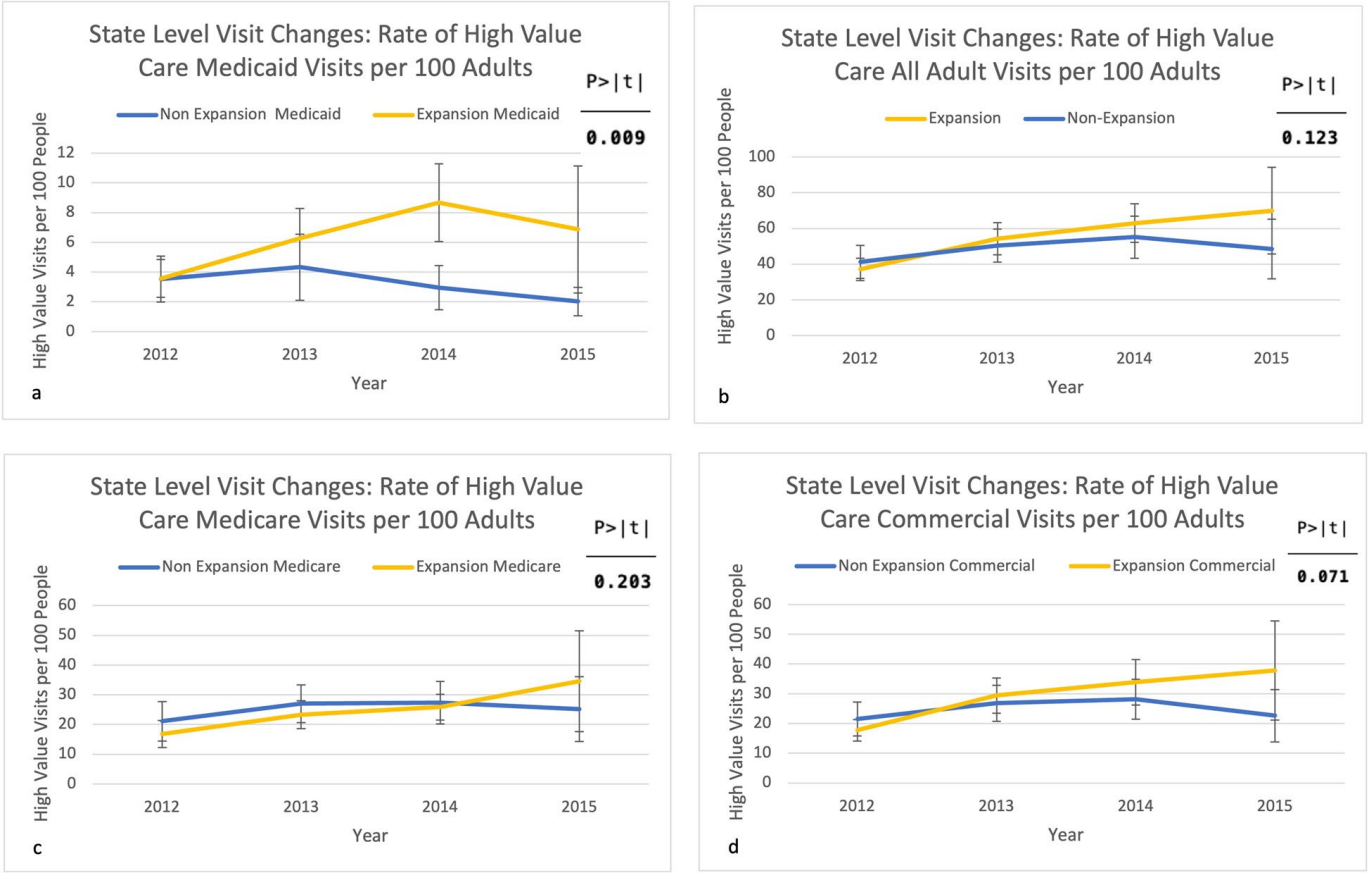
State-level high-value visit rate changes for Medicaid (**a**), all adult visits (**b**), Medicare (**c**), and Commercial (**d**)

We observed no significant changes in low-value care use in expansion versus non-expansion states (Fig. 2a and d). We observed no significant changes in high- or low-value care for all adult visits (Figs. 2b and 3b), Medicare (Figs. 2c and 3c), and commercially-insured visits (Figs. 2d and 3d). In contrast, high-value care in Medicaid visits did increase significantly in expansion states post-expansion by 4.3 visits per 100 adults (*p* = 0.009 95% CI 1.1–7.5, maintained after multiple-testing correction) (Fig. 3a). This was an increase unique to Medicaid as it was not observed in all adult visits, Medicare, nor commercially-insured visits.

## Discussion

Using a diverse sample of physician office visits across thirteen U.S. states, we observed that the ACA’s Medicaid expansion was associated with significant increases in Medicaid visits, and particularly new Medicaid visits, without unintended declines in visits among other insurance types, which is consistent with findings in Medicare [21], but not the commercial population [22]. This lack of substitution suggests that the U.S healthcare system had capacity to enhance access for Medicaid patients without sacrificing access to care for patients with other insurance types. Furthermore, we observed that quality on average did not deteriorate with the increased volume in visits for any insurer. Among visits by presumed new Medicaid enrollees, we found that Medicaid expansion was associated with greater receipt of high-quality care compared with non-expansion states.

The observed increase in visits following expanded insurance access has been well described; when people obtain health insurance, they utilize health care more [45, 46]. However, though several reports described increased insurance coverage as well as visit rates in community health centers and for older adults as a result of Medicaid expansion [1–14, 47, 48], this study is the first to quantify the change in office visit volume’s subsequent delivery of high and low-value care across all-payers. Expansion states may have been able to accommodate these increases by a number of mechanisms, including but not limited to: having excess capacity at baseline; increasing efficiency of care to allow for more patient visits (e.g. use of team-based care); improving workforce supply, in part by a migration of new physicians starting new practices from non-expansion states to expansion states because of Medicaid expansion [49].

This study builds on prior work and evaluates the association between Medicaid expansion and quality of care. Prior work has described frequent use of low-value care among Medicaid patients prior to Medicaid expansion [34, 50], though with focus on safety-net physicians compared to commercially-insured settings. In the context of no difference in rates of accepting Medicaid [51] and Medicaid patients remaining concentrated among a relatively small numbers of physicians following expansion [3], our analysis highlights that while high-value care increased, the use of low-value care did not grow despite increased clinical demand. Physicians were able to preserve the same measured quality of care to all patients following expansion and even increase to some degree high-value services to particularly new Medicaid patients, likely representing the underlying unmet need for this population prior to expansion. Given prior work showing worsening quality (such as increased inappropriate opioid and decreased statin prescribing) when clinician workload and cognitive demand increases, this finding is particularly notable [52, 53]. These findings are also concordant with prior evaluations demonstrating quality of care and time spent during visits is similar across insurance type [54, 55].

Our work is also consistent with prior work that has detailed improvements in quality of care at CHCs following Medicaid expansion [47, 56, 57]. While CHCs form a core component of healthcare delivery for the Medicaid population, our work provides a novel contribution by examining quality of care for Medicaid enrollees at non-CHC locations, which is often overlooked. Additionally, while outcomes such as control of chronic disease are important, we extend this prior work by examining composite measures that focus on process quality, which may mediate improvements in chronic disease outcomes.

Our study has several limitations. This observational cross-sectional (non-randomized) study cannot exclude unmeasured confounders and does not allow for longitudinal assessment of individual patients. Separately, NAMCS does not allow individual identification of patients newly enrolled in Medicaid, accordingly our new Medicaid patient population is the closest proxy to these individuals in our data. Additionally, there are two years pre- and post-Medicaid expansion for state-level estimates in NAMCS; evaluations of the effects of Medicaid expansion may benefit from further years of data. Furthermore, NAMCS only provides state-level estimates for a selected group of states each year, therefore we could not analyze Medicaid expansion across all US states and our results may have limited generalizability to less populated states. NAMCS has historically had large sample sizes, but relatively low response rates, particularly in the context of applying sampling weights, though to affect the data, non-response would have to occur differentially between expansion and non-expansion states. Additionally, our results may be biased towards the null as the expansion states in this analysis may have had different pre-expansion eligibility criteria compared to expansion states not included in this analysis and three of the expansion states began expanding coverage early. Lastly, our quality-of-care measures reflect a subset of high and low-value care and may misclassify true quality, though it is unlikely for misclassification to occur differentially between expansion and non-expansions states.

## Conclusion

Together, these findings suggest that following Medicaid expansion, the U.S. healthcare system enhanced access and improved high-quality care for millions of Americans enrolled in Medicaid, without observable reductions in access or quality for those enrolled in other insurance. However, the provision of low-value care continued at similar rates post-expansion, with important implications for future federal policy initiatives designed to improve the value of care.

## Supplementary Information

**Additional file 1.**

## Data Availability

Data used for this analysis is publicly available through NAMCS and US Census Bureau. Stata code for data management and analysis is provide in Supplementary material.

## Abbreviations

ACA: Affordable Care Act
ACEi: Angiotensin Converting Enzyme Inhibitor
ARB: Angiotensin Receptor Blocker
AZ: Arizona
CA: California
CHC: Community Health Center
DinD: difference-in-differences
FL: Florida
GA: Georgia
IL: Illinois
MA: Massachusetts
NAMCS: National Ambulatory Medical Care Survey
NC: North Carolina
NCHS: National Center for Health Statistics
NJ: New Jersey
NY: New York
OH: Ohio
TX: Texas
URI: Upper Respiratory Tract Infection
USPSTF: United Stated Preventive Services Task Force
VA: Virginia
WA: Washington
