# Supplementary material for: Evaluating the accessibility and value of U.S. ambulatory care among Medicaid expansion states and non-expansion states, 2012–2015
Source: BMC Health Serv Res. 2023 Jul 3;23:723. doi: 10.1186/s12913-023-09696-x (PMC10318663; doi:10.1186/s12913-023-09696-x)
Supplement: Supplementary file 1 — Additional file 1. [file 12913_2023_9696_MOESM1_ESM.docx]

**Appendix**

**Appendix Table 1: High Value and Low Value Measures**

| **Low-value services (7)** | **High-value services (10)** |
| --- | --- |
| Asx UTI Screen^28-34,40^ | Rx for ASA for CAD^28-35,40^ |
| Low Risk CV Screen^28-34,40^ | Statin for CAD^28-32,35,40^ |
| Abx for URI^28,30-37,40,41^ | BB for CAD^28-35,40^ |
| Opioid for headache^30,35,38-40^ | Rx for BB for CHF^28,30-32,34,35,40^ |
| Opioid for neck, back^30,35,39,40^ | Rx for ACEi/ARB in CHF^28-32,34,35,40^ |
| CT/MRI for headache ^30,35,38-41^ | Rx for AC for Afib^28-35,40^ |
| CT/MRI for neck/back pain^30,35,39-41^ | Rx for antiplt in cerebrovascular disease^28,30,31,34,40^ |
|  | Rx for statin in DM^28,30-32,34,40^ |
|  | Tx of depression^29,30,32-35,40^ |
|  | Tx for osteoporosis^28,30-32,34,40^ |

**ClinicalTrials.gov Observational Study Protocol:** <https://www.clinicaltrials.gov/ct2/show/NCT05319743?term=Medicaid+expansion%2C+value&type=Obsr&draw=2&rank=1>

**Stata Code for High Value and Low Value Measures**

***NAMCS Medicaid Expansion High and Low Value Measures***

***Stata 17.0**

***Data Management File**

***---------------------------------------------**

***Combining Data Files**

***---------------------------------------------**

***Prepare State Level Population Estimates**

***2012 State Level Population Estimates**

**clear**

**infile using "$path/data/R13079331.dct", using("$path/data/R13079331_SL040.txt")**

**drop NAME QNAME NATION STATE COUNTY**

**destring FIPS, replace force**

**keep if FIPS == 04 | FIPS ==06 | FIPS ==12 | FIPS ==13 | FIPS ==17 | FIPS ==25 | FIPS ==34 | FIPS ==36 | FIPS ==37 | FIPS ==39 | FIPS ==48 | FIPS ==51 | FIPS ==53**

**rename FIPS FIPSSTOFF**

**gen year = 2012**

**rename T006_001 TotPop2012**

**rename T006_002 Under18Pop2012**

**rename T006_003 Eighteento34Pop2012**

**rename T006_004 ThirtyFiveto64Pop2012**

**rename T006_005 Over65Pop2012**

**save "$path/data/2012_Population", replace**

***2013 State Level Population Estimates**

**clear**

**infile using "$path/data/R13079330.dct", using("$path/data/R13079330_SL040.txt")**

**drop NAME QNAME NATION STATE COUNTY**

**destring FIPS, replace force**

**keep if FIPS == 04 | FIPS ==06 | FIPS ==12 | FIPS ==13 | FIPS ==17 | FIPS ==25 | FIPS ==34 | FIPS ==36 | FIPS ==37 | FIPS ==39 | FIPS ==48 | FIPS ==51 | FIPS ==53**

**rename FIPS FIPSSTOFF**

**gen year = 2013**

**rename T006_001 TotPop2013**

**rename T006_002 Under18Pop2013**

**rename T006_003 Eighteento34Pop2013**

**rename T006_004 ThirtyFiveto64Pop2013**

**rename T006_005 Over65Pop2013**

**save "$path/data/2013_Population", replace**

***2014 State Level Population Estimates**

**clear**

**infile using "$path/data/R13079333.dct", using("$path/data/R13079333_SL040.txt")**

**drop NAME QNAME NATION STATE COUNTY**

**destring FIPS, replace force**

**keep if FIPS == 04 | FIPS ==06 | FIPS ==12 | FIPS ==13 | FIPS ==17 | FIPS ==25 | FIPS ==34 | FIPS ==36 | FIPS ==37 | FIPS ==39 | FIPS ==48 | FIPS ==51 | FIPS ==53**

**rename FIPS FIPSSTOFF**

**gen year = 2014**

**rename T006_001 TotPop2014**

**rename T006_002 Under18Pop2014**

**rename T006_003 Eighteento34Pop2014**

**rename T006_004 ThirtyFiveto64Pop2014**

**rename T006_005 Over65Pop2014**

**save "$path/data/2014_Population", replace**

***2015 State Level Population Estimates**

**clear**

**infile using "$path/data/R13079335.dct", using("$path/data/R13079335_SL040.txt")**

**drop NAME QNAME NATION STATE COUNTY**

**destring FIPS, replace force**

**keep if FIPS == 04 | FIPS ==06 | FIPS ==12 | FIPS ==13 | FIPS ==17 | FIPS ==25 | FIPS ==34 | FIPS ==36 | FIPS ==37 | FIPS ==39 | FIPS ==48 | FIPS ==51 | FIPS ==53**

**rename FIPS FIPSSTOFF**

**gen year = 2015**

**rename T006_001 TotPop2015**

**rename T006_002 Under18Pop2015**

**rename T006_003 Eighteento34Pop2015**

**rename T006_004 ThirtyFiveto64Pop2015**

**rename T006_005 Over65Pop2015**

**save "$path/data/2015_Population", replace**

***Preparing data for each year**

**foreach x in 2012 2013 2014 2015 {**

**use "$path/data/namcs`x'-stata.dta", clear**

***keep AGE FIPSSTOFF PAYMCAID PAYNOCHG PAYSELF PAYTYPER NOPAY PAYPRIV PAYMCARE PAYWKCMP PAYOTH PAYDK PATWTST NMEDCAID NSELFPAY NNOCHRGE SENBEFOR CPSUM PATWT PHYSWT CSTRATM**

**gen year=`x'**

***describe**

**save "$path/data/namcs`x'_small.dta", replace**

**}**

***Append data over 2012-2015**

**use "$path/data/namcs2012_small", clear**

**count**

**append using "$path/data/namcs2013_small"**

**count**

**append using "$path/data/namcs2014_small"**

**count**

**append using "$path/data/namcs2015_small"**

**count**

**save "$path/data/namcs2012_2015_small", replace**

***Merge NAMCS and State Population Estimates**

**use "$path/data/namcs2012_2015_small"**

**merge m:1 FIPSSTOFF year using "$path/data/2012_Population"**

**drop _merge**

**merge m:1 FIPSSTOFF year using "$path/data/2013_Population"**

**drop _merge**

**merge m:1 FIPSSTOFF year using "$path/data/2014_Population"**

**drop _merge**

**merge m:1 FIPSSTOFF year using "$path/data/2015_Population"**

**drop _merge**

**save "$path/data/CombinedNAMCS_POPULATION", replace**

***------------------------------------------------------------------------------**

***Generate Indicator Variables for States, Expansion and Subpopulations**

***------------------------------------------------------------------------------**

***State FIPS Codes (13)**

***04 Arizona**

***06 California**

***12 Florida**

***13 Georgia**

***17 Illinois**

***25 Massachusetts**

***34 New Jersey**

***36 New York**

***37 North Carolina**

***39 Ohio**

***48 Texas**

***51 Virginia**

***53 Washington**

***Generate State to be Included in Analysis Indicator Variable***

**gen State_tobe_Included = .**

**replace State_tobe_Included = 1 if FIPSSTOFF == 04 | FIPSSTOFF ==06 | FIPSSTOFF ==12 | FIPSSTOFF ==13 | FIPSSTOFF ==17 | FIPSSTOFF ==25 | FIPSSTOFF ==34 | FIPSSTOFF ==36 | FIPSSTOFF ==37 | FIPSSTOFF ==39 | FIPSSTOFF ==48 | FIPSSTOFF ==51 | FIPSSTOFF ==53**

**replace State_tobe_Included = 0 if State_tobe_Included ==.**

***Generate Adult Indicator Variable**

**recode AGE (min/17 = 0) (18/max = 1), gen(ADULT)**

***Generate Expansion Status Indicator Variable***

**gen expansion2014 = .**

**replace expansion2014 = 1 if FIPSSTOFF == 04 | FIPSSTOFF == 06 | FIPSSTOFF == 17 | FIPSSTOFF == 25 | FIPSSTOFF == 34 | FIPSSTOFF == 36 | FIPSSTOFF == 39 | FIPSSTOFF == 53**

**replace expansion2014 = 0 if FIPSSTOFF == 12 | FIPSSTOFF == 13 | FIPSSTOFF == 37 | FIPSSTOFF == 48 | FIPSSTOFF == 51**

***Generate Adult + 13 States Indicator Variable***

**gen Adults_13States = .**

**replace Adults_13States = 1 if State_tobe_Included == 1 & ADULT == 1**

**replace Adults_13States = 0 if Adults_13States == .**

***Generate Post-Expansion Indicator Variable***

**gen postexpansion = 0**

**replace postexpansion = 1 if year == 2014 | year == 2015**

***Generate Pre-Expansion Indicator to Assess Trends in Measures 2012 - 2013**

**gen preexpansion = 0**

**replace preexpansion = 1 if year == 2012 | year == 2013**

**gen PreExpansion_Adults_13States = .**

**replace PreExpansion_Adults_13States = 1 if State_tobe_Included == 1 & ADULT == 1 & preexpansion == 1**

**replace PreExpansion_Adults_13States = 0 if PreExpansion_Adults_13States == .**

***Generate Medicaid Patients Indicator Variable**

**gen McaidAdults_13States = 0**

**replace McaidAdults_13States = 1 if State_tobe_Included == 1 & ADULT == 1 & PAYMCAID == 1**

**gen PreExpan_McaidAdults_13States = 0**

**replace PreExpan_McaidAdults_13States = 1 if State_tobe_Included == 1 & ADULT == 1 & preexpansion == 1 & PAYMCAID == 1**

***Generate "New" Medicaid Patients Indicator Variable**

**gen NewMcaidAdults_13States = 0**

**replace NewMcaidAdults_13States = 1 if State_tobe_Included == 1 & ADULT == 1 & PAYMCAID == 1 & SENBEFOR == 2**

**gen PreExpan_NewMcaidAdults_13States = 0**

**replace PreExpan_NewMcaidAdults_13States = 1 if State_tobe_Included == 1 & ADULT == 1 & preexpansion == 1 & PAYMCAID == 1 & SENBEFOR == 2**

**gen AcceptNewMcaidAdults_13States = 0**

**replace AcceptNewMcaidAdults_13States = 1 if State_tobe_Included == 1 & ADULT == 1 & PAYMCAID == 1 & SENBEFOR == 2 & ACEPTNEW == 1 & NMEDCAID == 1**

**gen PreExAccNewMcaidAdults_13States = 0**

**replace PreExAccNewMcaidAdults_13States = 1 if AcceptNewMcaidAdults_13States == 1 & preexpansion == 1**

***Generate Uninsured Category (Combine Pay Type Self, No Charge/Charity)**

**gen PAYUNIN = 0**

**replace PAYUNIN = 1 if PAYSELF == 1 | PAYNOCHG == 1**

***---------------------------------------------**

***Operationalize LVC/HVC Measures**

***---------------------------------------------**

**/***

**LVC Measures**

**1L. Imaging for Low Back Pain**

**2L. Opioid for Low Back Pain**

**3L. Opioid for Headache**

**4L. Imaging for Headache**

**5L. Abx for URI**

**6L. GME ECG**

**7L. GME UA**

**HVC Measures**

**1H. AntiPlt for CAD**

**2H. BB for CAD**

**3H. Statin for CAD**

**4H. AC for Afib**

**5H. Statin for DM**

**6H. Antiplt CVD**

**7H. Tx for Depression**

**8H. BB for CHF**

**9H. ACE/ARB/ARNI for CHF**

**10H. Tx for Osteoprosis**

***/**

**{**

********-------------------------**

***1L. Imaging for Low Back Pain**

***2L. Opioid for Low Back Pain**

********-------------------------**

**{**

***Back Pain Inclusion Indicator Variable**

**gen BackPain_Inclusion = 0**

**foreach RFVvar of varlist RFV* {**

**replace BackPain_Inclusion = 1 if `RFVvar' == 19000 | `RFVvar' == 19050 | `RFVvar' == 19051 | `RFVvar' == 19052 | `RFVvar' == 19053 | `RFVvar' == 19100 | `RFVvar' == 19101 | `RFVvar' == 19102 | `RFVvar' == 19103 | `RFVvar' == 51100 | `RFVvar' == 51050 | `RFVvar' == 55150**

**}**

**/***

**RFV Inclusions**

**RFV 19000 Neck Symptoms**

**RFV 19050 Back Symptoms**

**RFV 19051 Back Symptoms, pain, ache, soreness, discomfort**

**RFV 19052 Back Symptoms, cramps, contractures, spasms**

**RFV 19053 Back Symptoms, limitation of movement, stiffness, tightness**

**RFV 19100 Low Back Symptoms**

**RFV 19101 Low Back Symptoms, pain, ache, soreness, discomfort**

**RFV 19102 Low Back Pain, cramps, contractures, spasms**

**RFV 19103 Low Back Symptoms, limitation of movement, stiffness, tightness**

**RFV 51100 Sprains and Strains, Back**

**RFV 51050 Sprains and Strains, Cervical Spine, Neck**

**RFV 55150 Injury, other and unspecified type, back**

***/**

**foreach DIAGvar of varlist DIAG*R {**

**replace BackPain_Inclusion = 1 if `DIAGvar' == 172010 | `DIAGvar' == 175610 | `DIAGvar' == 173850 | `DIAGvar' >= 172100 & `DIAGvar' <= 172190 | `DIAGvar' >= 172200 & `DIAGvar' <= 172290 | `DIAGvar' >= 172300 & `DIAGvar' <= 172380 | `DIAGvar' >= 172400 & `DIAGvar' <= 172490 | `DIAGvar' >= 173700 & `DIAGvar' <= 173790 | `DIAGvar' >= 173900 & `DIAGvar' <= 173940 | `DIAGvar' >= 184600 & `DIAGvar' <= 184690 | `DIAGvar' >= 184700 & `DIAGvar' <= 184790 | `DIAGvar' == 172390 | `DIAGvar' == 173840**

**}**

**/***

**DIAG Inclusions**

**DIAG 7201 Spinal Enthesopathy**

**DIAG 7239 Unspecified musculoskeletal disorders and symptoms referable to neck**

**DIAG 7384 Acquired spondylolisthesis**

**DIAG 7561 Congenital Anomalies of Spine**

**DIAG 7385 Other Acquired Deformity of Back or Spine**

**DIAG 7210 - 7219 CTL Spondylosis with/without Myelopathy, Kissing Spine, Ankylosing vertebral hyperostosis, Traumatic spondylopathy, Other allied disorders of spine, Spondylosis of unspecified site**

**DIAG 7220 - 7229 Displacement of CTL intervertebral disc without myelopathy, Schmorl's nodes, Degeneration of CTL intervertebral disc, Intervertebral disc disorder with myelopathy, Postlaminectomy syndrome, Other and unspecified disc disorder**

**DIAG 7230 - 7238 Spinal stenosis in cervical region, Cervicalgia, Cervicocranial syndrome, Brachial neuritis or radiculitis NOS, Torticollis, Panniculitis specified as affecting neck, Ossification of posterior longitudinal ligament in cervical region, Other syndromes affecting cervical region**

**DIAG 7240 - 7249 Spinal stenosis other than cervical, Pain in thoracic spine, Lumbago, Sciatica, Thoracic or lumbosacral neuritis or radiculitis, unspecified, Backache, unspecified, Disorders of sacrum, Disorders of coccyx, Other symptoms referable to back, Other unspecified back disorders**

**DIAG 7370 - 7379 Adolescent postural kyphosis, Kyphosis (acquired), Lordosis (acquired), Kyphoscoliosis and scoliosis, Curvature of spine associated with other conditions, Other curvatures of spine, Unspecified curvature of spine**

**DIAG 7390 - 7394 Nonallopathic lesions head region, Nonallopathic lesions cervical region, Nonallopathic lesions, thoracic region, Nonallopathic lesions lumbar region, Nonallopathic lesions, sacral region**

**DIAG 8460 - 8469 Sprain of lumbosacral (joint) (ligament), Sprain of sacroiliac ligament, Sprain of sacrospinatus (ligament), Sprain of sacrotuberous (ligament), Sprain of other specified sites of sacroiliac region, Sprain of unspecified site of sacroiliac region**

**DIAG 8470 - 8479 Sprain of neck CTLSC, Sprain of unspecified site of back**

***/**

***Back Pain Exclusion Indicator Variable**

**gen BackPain_Exclusion = 0**

**foreach RFVvar of varlist RFV* {**

**replace BackPain_Exclusion = 1 if `RFVvar' == 10100 | `RFVvar' == 10451 | `RFVvar' == 50100 | `RFVvar' == 12000 | `RFVvar' >= 21000 & `RFVvar' <= 21350 | `RFVvar' >= 12200 & `RFVvar' <= 12401 | `RFVvar' == 10050 | `RFVvar' == 16004 | `RFVvar' == 16550**

**}**

**/***

**RFV Exclusions**

**RFV 10100 Fever**

**RFV 10451 Recent Weight Loss**

**RFV 10050 Chills**

**RFV 16004 Incontinence of Stool**

**RFV 16550 Incontinence of Urine**

**RFV 50100 Fractures and Dislocations, Spinal Column**

**RFV 12000 Abnormal Involuntary Movements (Nervous System)**

**RFV 21000 - 21350 Cancer**

**RFV 12200 - 12401 Neuro System: Disturbances of Sensation, Vertigo/Dizziness, Weakness, Speech Disturbance, Other Symptoms referable to the nervous system**

***/**

**foreach DIAGvar of varlist DIAG*R {**

**replace BackPain_Exclusion = 1 if `DIAGvar' == 133630 | `DIAGvar' == 133680 | `DIAGvar' == 172270 | `DIAGvar' == 172280 | `DIAGvar' == 179940 | `DIAGvar' == 178060 | `DIAGvar' == 178300 | `DIAGvar' == 178320 | `DIAGvar' == 178079 | `DIAGvar' == 178080 | `DIAGvar' == 128590 | `DIAGvar' >= 178100 & `DIAGvar' <= 178199 | `DIAGvar' == 178321 | `DIAGvar' >= 114000 & `DIAGvar' <= 120999 | `DIAGvar' >= 180500 & `DIAGvar' <= 183000 | `DIAGvar' == 192611 | `DIAGvar' == 192612 | `DIAGvar' == 192900 | `DIAGvar' == 195200 | `DIAGvar' == 134460 | `DIAGvar' == 172920 | `DIAGvar' == 142100 | `DIAGvar' == 142110 | `DIAGvar' == 142190 | `DIAGvar' == 103800 | `DIAGvar' == 173000 | `DIAGvar' >= 185000 & `DIAGvar' <= 185400 | `DIAGvar' >= 190500 & `DIAGvar' <= 190900 | `DIAGvar' >= 195800 & `DIAGvar' <= 195900 | `DIAGvar' >= 130540 & `DIAGvar' <= 130570 | `DIAGvar' == 133690 | `DIAGvar' == 178830 | `DIAGvar' == 17876 | `DIAGvar' == 178839 | `DIAGvar' == 178834 | `DIAGvar' == 179070 | `DIAGvar' == 192520 | `DIAGvar' == 192900 | `DIAGvar' >= 195200 & `DIAGvar' <= 195290 | `DIAGvar' >= 173000 & `DIAGvar' <= 173090**

**}**

**/***

**DIAG Exclusions**

**DIAG 3363 Myelopathy in other diseases classified elsewhere**

**DIAG 3368 Other myelopathy**

**DIAG 7227 Intervertebral disc disorder with myelopathy**

**DIAG 7228 Postlaminectomy syndrome**

**DIAG 7994 Cachexia**

**DIAG 7806 Fever and other physiologic disturbances of temperature regulation**

**DIAG 7830 Anorexia**

**DIAG 7832 Abnormal loss of weight and underweight**

**DIAG 78079 Other malaise and fatigue**

**DIAG 7808 Generalized hyperhidrosis**

**DIAG 2859 Anemia, unspecified**

**DIAG 781 (7810-78199) Symptoms involving nervous and musculoskeletal systems**

**DIAG 78321 Loss of weight**

**DIAG 140 - 209 (140 - 20999) Malignant Neoplasms**

**DIAG 805 - 839 Fractures, Dislocations**

**********

**DIAG 92611 Crushing injury of back**

**DIAG 92612 Crushing injury of buttock**

**DIAG 929 Crushing injury of multiple and unspecified sites**

**DIAG 952 Spinal cord injury without evidence of spinal bone injury**

**DIAG 34460 Cauda equina syndrome without mention of neurogenic bladder**

**DIAG 7292 Neuralgia, neuritis, and radiculitis, unspecified**

**DIAG 4210 Acute and subacute bacterial endocarditis**

**DIAG 4211 Acute and subacute infective endocarditis in diseases classified elsewhere**

**DIAG 4219 Acute endocarditis, unspecified**

**DIAG 038 Septicemia**

**DIAG 01********

**DIAG 730 Osteomyelitis periostitis and other infections involving bone**

**DIAG 850 - 854 Concussion, cerebral laceration and contusion, intracranial injury/hemorrhage**

**DIAG 905 - 909 Late effects of musculoskeletal and connective tissue injuries, Late effects of injuries to skin and subcutaneous tissues, Late effects of injuries to the nervous system, Late effects of other and unspecified injuries, Late effects of other and unspecified external causes**

**DIAG 958 - 959 Certain early complications of trauma, Injury other and unspecified**

**DIAG 3054 - 3057 Nondependent sedative, hypnotic or anxiolytic abuse, Nondependent opioid abuse, Nondependent cocaine abuse, Nondependent amphetamine or related acting sympathomimetic abuse**

**DIAG 3369 Unspecified disease of spinal cord**

**DIAG 7883 Urinary Incontinence**

**DIAG 7876 Incontinence of Feces**

**DIAG 78839 Other Urinary Incontinence**

**DIAG 78834 Incontinence without sensory awareness**

**DIAG 7907 Baceremia**

**DIAG 9252 Crushing injury of neck**

**DIAG 9290 Crushing injury of multiple site, not elsewhere classified**

**DIAG 9520 - 9529 Spinal Cord Injury**

**DIAG 7300 - 7309 osteomyelitis categories**

***/**

***[CANCER] 2012 - 2015 Cancer**

**replace BackPain_Exclusion = 1 if CANCER == 1**

***Taking Opioid Indicator Variable**

**gen TakingOpioid = 0**

**foreach RXVar of varlist RX**V3C* {**

**replace TakingOpioid = 1 if `RXVar' == "060" | `RXVar' == "191"**

**}**

**/***

**/***

**Medications**

**RX Level 3 CAT ID 060 Narcotic Analgesics**

**RX Level 3 CAT ID 191 Narcotic Analgesics Combinations**

***/**

**foreach MEDVar of varlist MED** {**

**replace TakingOpioid = 1 if `MEDVar' ==**

**}**

**/*Medications**

**MED1 - MED10**

***/**

**foreach DRUGIDVar of varlist DRUGID** {**

**replace TakingOpioid = 1 if `DRUGIDVar' ==**

**}**

**/*Drug ID**

***/**

***/**

***CT or MRI Indicator Variable**

**gen CTorMRI = 0**

**replace CTorMRI = 1 if CATSCAN == 1 | MRI == 1**

***Imaging for Low Back Pain Indicator Variable***

**gen BackPainImaging_LowValue = 0**

**replace BackPainImaging_LowValue = 1 if BackPain_Inclusion == 1 & BackPain_Exclusion == 0 & CTorMRI == 1**

***Opioid for Low Back Pain Indicator Variable**

**gen BackPainOpioid_LowValue = 0**

**replace BackPainOpioid_LowValue = 1 if BackPain_Inclusion == 1 & BackPain_Exclusion == 0 & TakingOpioid == 1**

***Low Back Pain Potential Visit Indicator Variable***

**gen BackPainPotentialVisit = 0**

**replace BackPainPotentialVisit = 1 if BackPain_Inclusion == 1 & BackPain_Exclusion == 0**

**}**

********-------------------------**

***1H. AntiPlt for CAD**

********-------------------------**

**{**

***ASA CAD Inclusion Indicator Variable**

**gen ASACAD_inclusion = 0**

**foreach RFVvar of varlist RFV* {**

**replace ASACAD_inclusion = 1 if `RFVvar' == 25150**

**}**

**/***

**RFV Inclusions**

**RFV 25150 Ischemic Heart Disease**

***/**

**foreach DIAGvar of varlist DIAG*3D {**

**replace ASACAD_inclusion = 1 if `DIAGvar' >= "410" & `DIAGvar' <= "414"**

**}**

**/***

**DIAG Inclusions**

**DIAG 410 - 414 Ischemic Heart Disease**

***/**

***[CAD] 2014 - 2015 Coronary artery disease (CAD), ischemic heart disease (IHD), or history of myocardial infarction (MI)**

***[IHD] 2012 - 2103 Includes angina pectoris, coronary atherosclerosis, acute myocardial infarction, and other forms of ischemic heart disease.**

**replace ASACAD_inclusion = 1 if CAD == 1**

**replace ASACAD_inclusion = 1 if IHD == 1**

***ASA CAD Exclusion Indicator Variable**

**gen ASACAD_exclusion = 0**

**foreach RFVvar of varlist RFV* {**

**replace ASACAD_exclusion = 1 if `RFVvar' >= 15800 & `RFVvar' <= 15802 | `RFVvar' == 10700 | `RFVvar' == 17550 | `RFVvar' == 17403**

**}**

**/***

**RFV Exclusions**

**RFV 15800 - 15802 Gastrointestinal Bleeding**

**RFV 10700 Bleeding, multiple or unspecified sites**

**RFV 17550 Uterine and vaginal bleeding**

**RFV 17403 Abnormal material, including clots**

***/**

**foreach DIAGvar of varlist DIAG*3D {**

**replace ASACAD_exclusion = 1 if `DIAGvar' == "578" | `DIAGvar' == "535" | `DIAGvar' >= "430" & `DIAGvar' <= "432" | `DIAGvar' == "626"**

**}**

**/***

**DIAG Exclusions**

**DIAG 578 Gastrointestinal Bleeding**

**DIAG 535 Gastritis**

**DIAG 430 - 432 CNS Bleeding**

**DIAG 626 Disorders of menstruation and other abnormal bleeding from female genital tract**

***/**

***Taking AntiPlt Indicator Variable**

**gen TakingAntiPlt = 0**

**foreach RXVar of varlist RX**V3C* {**

**replace TakingAntiPlt = 1 if `RXVar' == "062"**

**}**

**foreach RXVar of varlist RX**V2C* {**

**replace TakingAntiPlt = 1 if `RXVar' == "083"**

**}**

**/***

**Medications**

**RX Level 3 CAT ID 062 Salicylates**

**RX Level 2 CAT ID 083 Antiplatelet Agents**

***/**

**foreach MEDVar of varlist MED** {**

**replace TakingAntiPlt = 1 if `MEDVar' == 2805 | `MEDVar' == 40185 | `MEDVar' == 97174**

**}**

**/*Medications**

**MED1 - MED10**

**2805 Generic Aspirin**

**40185 Bayer Aspirin**

**97174 Baby Aspirin**

**10975 Ecotrin**

***/**

**foreach DRUGIDVar of varlist DRUGID** {**

**replace TakingAntiPlt = 1 if `DRUGIDVar' == "d00170" & `DRUGIDVar' != "d00842"**

**}**

**/*Drug ID**

**d00170 Aspirin**

***/**

**foreach DRUGIDVar of varlist DRUGID** {**

**replace TakingAntiPlt = 0 if `DRUGIDVar' == "d00842"**

**}**

**/*Drug ID**

**d00842 SALSALATE (Do not include)**

***/**

***ASA Low Value Indicator Variable**

**gen ASACAD_lowvalue = 0**

**replace ASACAD_lowvalue = 1 if ASACAD_inclusion == 1 & ASACAD_exclusion == 0 & TakingAntiPlt == 0**

***ASA High Value Indicator Variable**

**gen ASACAD_highvalue = 0**

**replace ASACAD_highvalue = 1 if ASACAD_inclusion == 1 & ASACAD_exclusion == 0 & TakingAntiPlt == 1**

***ASA High Value Visit Potential**

**gen ASACADPotentialVisit = 0**

**replace ASACADPotentialVisit = 1 if ASACAD_inclusion == 1 & ASACAD_exclusion == 0**

**}**

********-------------------------**

***2H. BB for CAD**

********-------------------------**

**{**

***BB CAD Inclusion Indicator Variable**

**gen BBCAD_inclusion = 0**

**foreach RFVvar of varlist RFV* {**

**replace BBCAD_inclusion = 1 if `RFVvar' == 25150**

**}**

**/***

**RFV Inclusions**

**RFV 25150 Ischemic Heart Disease**

***/**

**foreach DIAGvar of varlist DIAG*3D {**

**replace BBCAD_inclusion = 1 if `DIAGvar' >= "410" & `DIAGvar' <= "414"**

**}**

**/***

**DIAG Inclusions**

**DIAG 410 - 414 Ischemic Heart Disease**

***/**

***[CAD] 2014 - 2015 Coronary artery disease (CAD), ischemic heart disease (IHD), or history of myocardial infarction (MI)**

***[IHD] 2012 - 2103 Includes angina pectoris, coronary atherosclerosis, acute myocardial infarction, and other forms of ischemic heart disease.**

**replace ASACAD_inclusion = 1 if CAD == 1**

**replace ASACAD_inclusion = 1 if IHD == 1**

***BB CAD Exclusion Indicator Variable**

**gen BBCAD_exclusion = 0**

**foreach RFVvar of varlist RFV* {**

**replace BBCAD_exclusion = 1 if `RFVvar' == 26250 | `RFVvar' == 26200**

**}**

**/***

**RFV Exclusions**

**RFV 26250 Asthma**

**RFV 26200 Emphysema**

***/**

**foreach DIAGvar of varlist DIAG*3D {**

**replace BBCAD_exclusion = 1 if `DIAGvar' >= "491" & `DIAGvar' <= "493"**

**}**

**foreach DIAGvar of varlist DIAG*R {**

**replace BBCAD_exclusion = 1 if `DIAGvar' >= 142600 & `DIAGvar' <= 142660 | `DIAGvar' == 142781**

**}**

**/***

**DIAG Exclusions**

**DIAG 491 - 493 Chronic Bronchitis, Emphysema, Asthma**

**DIAG 4260 - 4266 Conduciton Disorders**

**DIAG 42781 Sinoatrial Node Dysfunction**

***/**

***[ASTHMA] 2012 - 2015 Asthma**

***[COPD] 2012 - 2015 COPD**

**replace BBCAD_exclusion = 1 if ASTHMA == 1**

**replace BBCAD_exclusion = 1 if COPD == 1**

***Taking BB Indicator Variable**

**gen TakingBB = 0**

**foreach RXVar of varlist RX**V2C* {**

**replace TakingBB = 1 if `RXVar' == "047"**

**}**

**/*Medications**

**RX Level 2 CAT ID 047 beta-adrenergic blocking agents**

**MED1 - MED10**

**94053 Beta Blocker**

**19218 METOPROLOL**

**01022 METOPROLOL TARTRATE**

**03272 METOPROLOL SUCCINATE**

**08089 METOPROLOL/HYDROCHLOROTHIAZIDE**

**98008 CARVEDILOL**

**00161 BISOPROLOL**

**04023 BISOPROLOL/HCTZ**

**04320 ATENOLOL/CHLORTHALIDONE**

**07436 HYDROCHLOROTHIAZIDE-ATENOLOL**

**08592 NEBIVOLOL**

**09589 PROPRANOLOL ER**

**42985 PROPRANOLOL**

**42990 PROPRANOLOL W/HCTZ**

**61245 PROPRANOLOL HCL**

**91063 ATENOLOL**

**92138 NADOLOL**

**93124 PINDOLOL**

**93446 BETAXOLOL**

**94173 ACEBUTOLOL**

**95186 ESMOLOL**

***/**

**foreach DRUGIDVar of varlist DRUGID** {**

**replace TakingBB = 1 if `DRUGIDVar' == "d00134" | `DRUGIDVar' == "d03264" | `DRUGIDVar' == "d03847" | `DRUGIDVar' == "d03744" | `DRUGIDVar' == "d05265" | `DRUGIDVar' == "d00004" | `DRUGIDVar' == "d00018" | `DRUGIDVar' == "d00032" | `DRUGIDVar' == "d00137" | `DRUGIDVar' == "d00224" | `DRUGIDVar' == "d00709" | `DRUGIDVar' == "d03258" | `DRUGIDVar' == "d03261"**

**}**

**/*Drug ID**

**d00134 Metoprolol**

**d03264 HCTZ-Metoprolol**

**d03847 CARVEDILOL**

**d03744 BISOPROLOL-HYDROCHLOROTHIAZIDE**

**d05265 NEBIVOLOL**

**d00004 ATENOLOL**

**d00018 NADOLOL**

**d00032 PROPRANOLOL**

**d00137 PINDOLOL**

**d00224 ESMOLOL**

**d00709 BISOPROLOL**

**d03258 ATENOLOL-CHLORTHALIDONE**

**d03261 HYDROCHLOROTHIAZIDE-PROPRANOLOL**

***/**

***BB CAD Low Value Indicator Variable**

**gen BBCAD_lowvalue = 0**

**replace BBCAD_lowvalue = 1 if BBCAD_inclusion == 1 & BBCAD_exclusion == 0 & TakingBB == 0**

***BB CAD High Value Indicator Variable**

**gen BBCAD_Highvalue = 0**

**replace BBCAD_Highvalue = 1 if BBCAD_inclusion == 1 & BBCAD_exclusion == 0 & TakingBB == 1**

***BB CAD Potential Visits Indicator Variable***

**gen BBCADHighValuePotential = 0**

**replace BBCADHighValuePotential = 1 if BBCAD_inclusion == 1 & BBCAD_exclusion == 0**

**}**

********-------------------------**

***3H. Statin for CAD**

********-------------------------**

**{**

***Statin CAD Inclusion Indicator Variable**

**gen StatinCAD_inclusion = 0**

**foreach RFVvar of varlist RFV* {**

**replace StatinCAD_inclusion = 1 if `RFVvar' == 25150**

**}**

**/***

**RFV Inclusions**

**RFV 25150 Ischemic Heart Disease**

***/**

**foreach DIAGvar of varlist DIAG*3D {**

**replace StatinCAD_inclusion = 1 if `DIAGvar' >= "410" & `DIAGvar' <= "414"**

**}**

**/***

**DIAG Inclusions**

**DIAG 410 - 414 Ischemic Heart Disease**

***/**

***[CAD] 2014 - 2015 Coronary artery disease (CAD), ischemic heart disease (IHD), or history of myocardial infarction (MI)**

***[IHD] 2012 - 2103 Includes angina pectoris, coronary atherosclerosis, acute myocardial infarction, and other forms of ischemic heart disease.**

**replace ASACAD_inclusion = 1 if CAD == 1**

**replace ASACAD_inclusion = 1 if IHD == 1**

***Statin CAD Exclusion Indicator Variable**

**gen StatinCAD_exclusion = 0**

**foreach RFVvar of varlist RFV* {**

**replace StatinCAD_exclusion = 1 if `RFVvar' == 23200**

**}**

**/***

**RFV Exclusions**

**RFV 23200 Alcoholism**

***/**

**foreach DIAGvar of varlist DIAG*3D {**

**replace StatinCAD_exclusion = 1 if `DIAGvar' >= "570" & `DIAGvar' <= "573" |`DIAGvar' == "303" | `DIAGvar' == "305"**

**}**

**foreach DIAGvar of varlist DIAG*R {**

**replace StatinCAD_exclusion = 1 if `DIAGvar' == 135940**

**}**

**/***

**DIAG Exclusions**

**DIAG 303 EtOH Dependence**

**DIAG 3050 (Change to 305 3 digit EtOH Abuse)**

**DIAG 570 - 573 Liver Disease**

**DIAG 3594 Toxic Myopathy**

***/**

***Taking Statin Indicator Variable**

**gen TakingStatin = 0**

**foreach RXVar of varlist RX**V3C* {**

**replace TakingStatin = 1 if `RXVar' == "173"**

**}**

**/***

**Medications**

**RX Level 3 CAT ID 173 HMG-CoA reductase inhibitors**

***/**

**foreach MEDVar of varlist MED** {**

**replace TakingStatin = 1 if `MEDVar' == 04679 | `MEDVar' == 05141 | `MEDVar' == 08161 | `MEDVar' == 09869 | `MEDVar' == 12164 | `MEDVar' == 91088 | `MEDVar' == 93237 | `MEDVar' == 95092 | `MEDVar' == 95140 | `MEDVar' == 97157**

**}**

**/*MED1 - MED10**

**04679 STATINS**

**05141 AVASTATIN**

**08161 ROSUVASTATIN**

**09869 AMLODIPINE/ATORVASTATIN**

**12164 LOVASTATIN-NIACIN**

**91088 LOVASTATIN**

**93237 SIMVASTATIN**

**95092 PRAVASTATIN**

**95140 FLUVASTATIN**

**97157 ATORVASTATIN**

***/**

**foreach DRUGIDVar of varlist DRUGID** {**

**replace TakingStatin = 1 if `DRUGIDVar' == "d00280" | `DRUGIDVar' == "d00348" | `DRUGIDVar' == "d00746" | `DRUGIDVar' == "d03183" | `DRUGIDVar' == "d04105" | `DRUGIDVar' == "d04851" | `DRUGIDVar' == "d04787" | `DRUGIDVar' == "d05048" | `DRUGIDVar' == "d05348" | `DRUGIDVar' == "d07110" | `DRUGIDVar' == "d08089" | `DRUGIDVar' == "d07637"**

**}**

**/*Drug ID**

**d00280 LOVASTATIN**

**d00348 PRAVASTATIN**

**d00746 SIMVASTATIN**

**d03183 FLUVASTATIN**

**d04105 ATORVASTATIN**

**d04851 ROSUVASTATIN**

**d04787 LOVASTATIN-NIACIN**

**d05048 AMLODIPINE-ATORVASTATIN**

**d05348 EZETIMIBE-SIMVASTATIN**

**d07110 NIACIN-SIMVASTATIN**

**d08089 ATORVASTATIN-EZETIMIBE**

**d07637 PITAVASTATIN**

***/**

***Statin CAD Low Value Indicator Variable**

**gen StatinCAD_lowvalue = 0**

**replace StatinCAD_lowvalue = 1 if StatinCAD_inclusion == 1 & StatinCAD_exclusion == 0 & TakingStatin == 0**

***Statin CAD High Value Indicator Variable**

**gen StatinCAD_Highvalue = 0**

**replace StatinCAD_Highvalue = 1 if StatinCAD_inclusion == 1 & StatinCAD_exclusion == 0 & TakingStatin == 1**

***Statin CAD High Value Visit Potential Indicator Variable***

**gen StatinCADHighValuePotential = 0**

**replace StatinCADHighValuePotential = 1 if StatinCAD_inclusion == 1 & StatinCAD_exclusion == 0**

**}**

********-------------------------**

***4H. AC for Afib**

********-------------------------**

**{**

***AC Afib Inclusion Indicator Variable**

**gen ACAfib_inclusion = 0**

**foreach DIAGvar of varlist DIAG*R {**

**replace ACAfib_inclusion = 1 if `DIAGvar' == 142731 | `DIAGvar' == 142732**

**}**

**/***

**DIAG Inclusions**

**DIAG 42731 Afib**

**DIAG 42732 Aflutter**

***/**

***AC Afib Exclusion Indicator Variable**

**gen ACAfib_exclusion = 0**

**foreach RFVvar of varlist RFV* {**

**replace ACAfib_exclusion = 1 if `RFVvar' >= 15800 & `RFVvar' <= 15802 | `RFVvar' == 23200 | `RFVvar' == 11500 | `RFVvar' == 23210 | `RFVvar' == 10950 | `RFVvar' == 12050 | `RFVvar' == 22550 | `RFVvar' == 10700 | `RFVvar' == 17550 | `RFVvar' == 17403**

**}**

**/***

**RFV Exclusions**

**RFV 15800 - 15802 Gastrointestinal Bleeding**

**23200 Alcoholism**

**11500 Abnormal Drug Usage**

**23210 Drug Dependence*** (Drug addition, nicorette dependency)**

**10950 Disorders of Motor Functions (Gait Disorder)**

**12050 Convulsions**

**22550 Other Diseases of Blood and Blood Forming Organs (Bleeding)**

**RFV 10700 Bleeding, multiple or unspecified sites**

**RFV 17550 Uterine and vaginal bleeding**

**RFV 17403 Abnormal material, including clots**

***/**

**foreach DIAGvar of varlist DIAG*3D {**

**replace ACAfib_exclusion = 1 if `DIAGvar' == "578" |`DIAGvar' == "535" | `DIAGvar' == "303" | `DIAGvar' == "304" | `DIAGvar' == "305" | `DIAGvar' == "345" | `DIAGvar' == "191" | `DIAGvar' == "287" | `DIAGvar' >= "430" & `DIAGvar' <= "432" | `DIAGvar' == "290" | `DIAGvar' == "626"**

**}**

**foreach DIAGvar of varlist DIAG*R {**

**replace ACAfib_exclusion = 1 if `DIAGvar' >= 178031 & `DIAGvar' <= 178039 | `DIAGvar' == 123960 | `DIAGvar' == 178120 | `DIAGvar' >= 129410 & `DIAGvar' <= 129421**

**}**

**/***

**DIAG Exclusions**

**DIAG 578 Gastrointestinal Bleeding**

**DIAG 535 Gastritis**

**DIAG 303 Alcohol Dependence Syndrome**

**DIAG 3050 Alcohol Abuse (Change to 305 3 Digit)**

**DIAG 3052 - 3059 (Change to 305 3 Digit)**

**DIAG 305 Nondependent abuse of drugs**

**DIAG 304 Drug Dependence**

**DIAG 78031 - 78039 Convulsions**

**DIAG 345 Epilepsy**

**DIAG 2396 Brain Neoplasm NOS**

**DIAG 191 Malignant Neoplasm of the Brain**

**DIAG 287 Purpura and Other Hemorrhagic Conditions**

**DIAG 7812 Abnormality of Gait**

**DIAG 2941 - 29421 Persistent mental disorders due to conditions classified elsewhere**

**DIAG 430 - 432 CNS Bleeding**

**DIAG 290 Dementia**

**DIAG 626 Disorders of menstruation and other abnormal bleeding from female genital tract**

***/**

***Taking AC Indicator Variable**

**gen TakingAC = 0**

**foreach RXVar of varlist RX**V2C* {**

**replace TakingAC = 1 if `RXVar' == "082"**

**}**

**replace TakingAC = 1 if TakingAntiPlt == 1**

**/***

**Medications**

**RX Level 2 CAT ID 082 Anticoagulants (heparins, coumarins and indandiones, thrombin inhibitors, factor Xa inhibitors)**

***/**

**foreach MEDVar of varlist MED** {**

**replace TakingAC = 1 if `MEDVar' == 09958 | `MEDVar' == 14002 | `MEDVar' == 34775 | `MEDVar' == 04351 | `MEDVar' == 07930 | `MEDVar' == 08284 | `MEDVar' == 10356 | `MEDVar' == 11393 | `MEDVar' == 09177 | `MEDVar' == 13009 | `MEDVar' == 94117 | `MEDVar' == 00198 | `MEDVar' == 03318 | `MEDVar' == 12216 | `MEDVar' == 14240**

**}**

**/*MED1 - MED10**

**09958 RIVAROXABAN**

**14002 APIXABAN**

**34775 WARFARIN**

**04351 JANTOVEN**

**07930 COUMADIN**

**08284 DABIGATRAN**

**10356 PRADAXA**

**11393 XARELTO**

**09177 BIVALIRUDIN**

**13009 ELIQUIS**

**94117 LOVENOX**

**00198 ENOXAPARIN SODIUM**

**03318 HEPARIN SODIUM**

**12216 HEPARIN PORCINE**

**14240 HEPARIN**

***/**

**foreach DRUGIDVar of varlist DRUGID** {**

**replace TakingAC = 1 if `DRUGIDVar' == "c00082" | `DRUGIDVar' == "d07356" | `DRUGIDVar' == "d07804" | `DRUGIDVar' == "n09189" | `DRUGIDVar' == "d00022" | `DRUGIDVar' == "d07137" | `DRUGIDVar' == "n08081" | `DRUGIDVar' == "d04744" | `DRUGIDVar' == "d03041" | `DRUGIDVar' == "d00252"**

**}**

**/*Drug ID**

**c00082 ANTICOAGULANTS**

**d07356 RIVAROXABAN**

**d07804 APIXABAN**

**n09189 RIVAROXABAN**

**d00022 WARFARIN**

**d07137 DABIGATRAN**

**n08081 DABIGATRAN**

**d04744 BIVALIRUDIN**

**d03041 ENOXAPARIN**

**d00252 HEPARIN**

***/**

***AC Afib Low Value Indicator Variable**

**gen ACAfib_lowvalue = 0**

**replace ACAfib_lowvalue = 1 if ACAfib_inclusion == 1 & ACAfib_exclusion == 0 & TakingAC == 0**

***AC Afib High Value Indicator Variable**

**gen ACAfib_Highvalue = 0**

**replace ACAfib_Highvalue = 1 if ACAfib_inclusion == 1 & ACAfib_exclusion == 0 & TakingAC == 1**

***AC Afib High Value Potential Visit Indicator Variable**

**gen ACAfibPotentialVisit = 0**

**replace ACAfibPotentialVisit = 1 if ACAfib_inclusion == 1 & ACAfib_exclusion == 0**

**}**

********-------------------------**

***5H. Statin for DM**

********-------------------------**

**{**

***Statin DM Inclusion Indicator Variable**

**gen StatinDM_inclusion = 0**

**foreach RFVvar of varlist RFV* {**

**replace StatinDM_inclusion = 1 if `RFVvar' == 22050**

**}**

**/***

**RFV Inclusions**

**RFV 22050 DM**

***/**

**foreach DIAGvar of varlist DIAG*3D {**

**replace StatinDM_inclusion = 1 if `DIAGvar' >= "249" & `DIAGvar' <= "250"**

**}**

**/***

**DIAG Inclusions**

**DIAG 249 Secondary DM**

**DIAG 250 DM**

***/**

***DM 2014 - 2015 == [DIABTYP1] Diabetes mellitus (DM), type I [DIABTYP2] Diabetes mellitus (DM), type II [DIABTYP0] Diabetes mellitus (DM), type unspecified**

***[DIABETES] 2012 - 2013 Diabetes**

**replace StatinDM_inclusion = 1 if DIABTYP0 == 1 | DIABTYP1 == 1 | DIABTYP2 == 1**

**replace StatinDM_inclusion = 1 if DIABETES == 1**

***Statin DM Exclusion Indicator Variable**

**gen StatinDM_exclusion = 0**

**foreach RFVvar of varlist RFV* {**

**replace StatinDM_exclusion = 1 if `RFVvar' == 23200**

**}**

**/***

**RFV Exclusions**

**RFV 23200 Alcoholism**

***/**

**foreach DIAGvar of varlist DIAG*3D {**

**replace StatinDM_exclusion = 1 if `DIAGvar' >= "570" & `DIAGvar' <= "573" |`DIAGvar' == "303" | `DIAGvar' == "305"**

**}**

**foreach DIAGvar of varlist DIAG*R {**

**replace StatinCAD_exclusion = 1 if `DIAGvar' == 135940**

**}**

**/***

**DIAG Exclusions**

**DIAG 303 EtOH Dependence**

**DIAG 3050 (Change to 305 3 digit EtOH Abuse)**

**DIAG 570 - 573 Liver Disease**

**DIAG 3594 Toxic Myopathy**

***/**

***Statin DM Low Value Indicator Variable**

**gen StatinDM_lowvalue = 0**

**replace StatinDM_lowvalue = 1 if StatinDM_inclusion == 1 & StatinDM_exclusion == 0 & TakingStatin == 0**

***Statin DM High Value Indicator Variable**

**gen StatinDM_Highvalue = 0**

**replace StatinDM_Highvalue = 1 if StatinDM_inclusion == 1 & StatinDM_exclusion == 0 & TakingStatin == 1**

***Statin DM Potential Visit Indicator Variable**

**gen StatinDMPotentialVisit = 0**

**replace StatinDMPotentialVisit = 1 if StatinDM_inclusion == 1 & StatinDM_exclusion == 0**

**}**

********-------------------------**

***6H. AntiPlt for CVD**

********-------------------------**

**{**

***AntiPlt CVD Inclusion Indicator Variable**

**gen AntiPltCVD_inclusion = 0**

**foreach RFVvar of varlist RFV* {**

**replace AntiPltCVD_inclusion = 1 if `RFVvar' == 25250**

**}**

**/***

**RFV Inclusions**

**RFV 25250 CVD**

***/**

**foreach DIAGvar of varlist DIAG*3D {**

**replace AntiPltCVD_inclusion = 1 if `DIAGvar' >= "433" & `DIAGvar' <= "438"**

**}**

**/***

**DIAG Inclusions**

**DIAG 433 - 438 Ischemic Heart Disease (Occlusion and stenosis of precerebral arteries, Occlusion of cerebral arteries, TIA, CVA, Other and ill-defined cerebrovascular disease, Late effects of cerebrovascular disease)**

***/**

***[CEBVD] 2012 - 2015 Cerebrovascular disease/History of stroke (CVA) or transient ischemic attack (TIA)**

**replace AntiPltCVD_inclusion = 1 if CEBVD == 1**

***AntiPltCVD CVD Exclusion Indicator Variable**

**gen AntiPltCVD_exclusion = 0**

**foreach RFVvar of varlist RFV* {**

**replace AntiPltCVD_exclusion = 1 if `RFVvar' >= 15800 & `RFVvar' <= 15802 | `RFVvar' == 10700 | `RFVvar' == 17550 | `RFVvar' == 17403**

**}**

**/***

**RFV Exclusions**

**RFV 15800 - 15802 Gastrointestinal Bleeding**

**RFV 10700 Bleeding, multiple or unspecified sites**

**RFV 17550 Uterine and vaginal bleeding**

**RFV 17403 Abnormal material, including clots**

***/**

**foreach DIAGvar of varlist DIAG*3D {**

**replace AntiPltCVD_exclusion = 1 if `DIAGvar' == "578" | `DIAGvar' == "535" | `DIAGvar' >= "430" & `DIAGvar' <= "432" | `DIAGvar' == "626"**

**}**

**/***

**DIAG Exclusions**

**DIAG 578 Gastrointestinal Bleeding**

**DIAG 535 Gastritis**

**DIAG 430 - 432 CNS Bleeding**

**DIAG 626 Disorders of menstruation and other abnormal bleeding from female genital tract**

***/**

***AntiPlt CVD Low Value Indicator Variable**

**gen AntiPltCVD_lowvalue = 0**

**replace AntiPltCVD_lowvalue = 1 if AntiPltCVD_inclusion == 1 & AntiPltCVD_exclusion == 0 & TakingAntiPlt == 0**

***AntiPlt CVD High Value Indicator Variable**

**gen AntiPltCVD_Highvalue = 0**

**replace AntiPltCVD_Highvalue = 1 if AntiPltCVD_inclusion == 1 & AntiPltCVD_exclusion == 0 & TakingAntiPlt == 1**

***AntiPlt CVD Potential Visit Indicator Variable**

**gen AntiPltCVDPotentialVisit = 0**

**replace AntiPltCVDPotentialVisit = 1 if AntiPltCVD_inclusion == 1 & AntiPltCVD_exclusion == 0**

**}**

********-------------------------**

***7H. Depression Tx**

********-------------------------**

**{**

***Depression Inclusion Indicator Variable**

**gen Dep_inclusion = 0**

**foreach RFVvar of varlist RFV* {**

**replace Dep_inclusion = 1 if `RFVvar' == 11100**

**}**

**/***

**RFV Inclusions**

**RFV 11100 Depression Symptom**

***/**

**foreach DIAGvar of varlist DIAG*3D {**

**replace Dep_inclusion = 1 if `DIAGvar' == "311"**

**}**

**foreach DIAGvar of varlist DIAG*R {**

**replace Dep_inclusion = 1 if `DIAGvar' >= 129620 & `DIAGvar' <= 129633**

**}**

**/***

**DIAG Inclusions**

**DIAG 311 Depressive disorder NEC**

**DIAG 2962 - 2963 (extend to 296.33)**

***/**

***[DEPRN] 2012 - 2105 Depression**

**replace Dep_inclusion = 1 if DEPRN == 1**

***Depression Tx Indicator Variable**

**gen DepTx = 0**

**foreach RXVar of varlist RX**V2C* {**

**replace DepTx = 1 if `RXVar' == "249"**

**}**

**/***

**Medications**

**RX Level 2 CAT ID 249 Antidepressants**

***/**

***[MENTAL] 2012 - 2015 Mental health counseling, excluding psychotherapy**

**replace DepTx = 1 if MENTAL == 1**

***[PSYCHOTH] 2012 - 2015 Psychotherapy**

**replace DepTx = 1 if PSYCHOTH == 1**

***Depression Tx Low Value Indicator Variable***

**gen DepTx_lowvalue = 0**

**replace DepTx_lowvalue = 1 if Dep_inclusion == 1 & DepTx == 0**

***Depression Tx High Value Indicator Variable***

**gen DepTx_highvalue = 0**

**replace DepTx_highvalue = 1 if Dep_inclusion == 1 & DepTx == 1**

**}**

********-------------------------**

***4L. Opioid for Headache**

***5L. Imaging for Headache**

********-------------------------**

**{**

***Opioid Headache Inclusion Indicator Variable**

**gen OpioidHA_inclusion = 0**

**foreach RFVvar of varlist RFV* {**

**replace OpioidHA_inclusion = 1 if `RFVvar' == 12100 | `RFVvar' == 23650**

**}**

**/***

**RFV Inclusions**

**RFV 12100 Headache, pain in head**

**RFV 23650 Migraine Headache**

***/**

**foreach DIAGvar of varlist DIAG*3D {**

**replace OpioidHA_inclusion = 1 if `DIAGvar' == "346" | `DIAGvar' == "339"**

**}**

**foreach DIAGvar of varlist DIAG*R {**

**replace OpioidHA_inclusion = 1 if `DIAGvar' == 178400 | `DIAGvar' == 130781**

**}**

**/***

**DIAG Inclusions**

**DIAG 7840 Headache**

**DIAG 346 Migraine**

**DIAG 30781 Tension Headache**

**DIAG 339 Other Headache Syndromes**

***/**

***Opioid Headache Exclusion Indicator Variable**

**gen OpioidHA_exclusion = 0**

**foreach RFVvar of varlist RFV* {**

**replace OpioidHA_exclusion = 1 if `RFVvar' == 10100 | `RFVvar' == 12202 | `RFVvar' == 12203 | `RFVvar' == 12300 | `RFVvar' == 12350 | `RFVvar' == 12352 | `RFVvar' == 58420 | `RFVvar' == 55050 | `RFVvar' == 17900 | `RFVvar' == 27350 | `RFVvar' == 20151 | `RFVvar' == 23600 | `RFVvar' == 50050 | `RFVvar' >= 21000 & `RFVvar' <= 21350**

**}**

**/***

**RFV Exclusions**

**RFV 10100 Fever**

**RFV 12202 Increased sensation (hyperesthesia)**

**RFV 12203 Abnormal sensation (paresthesia)**

**RFV 12300 Weakness (neurologic)**

**RFV 12350 Disorders of speech, speech disturbance**

**RFV 12352 Slurring**

**RFV 58420 Altered level of consciousness, NOS**

**RFV 55050 Injury, orht and unspecified type (Head, neck, and face)**

**RFV 17900 Problems of pregnancy**

**RFV 27350 Diagnosed complications of pregnancy and puerperium**

**RFV 20151 History of positive HIV test findings**

**RFV 23600 Epilepsy**

**RFV 50050 Fractures and Dislocations (Head and Face)**

**RFV 21000 - 21350 Cancer**

***/**

**foreach DIAGvar of varlist DIAG*3D {**

**replace OpioidHA_exclusion = 1 if `DIAGvar' >= "801" & `DIAGvar' <= "804" | `DIAGvar' >= "850" & `DIAGvar' <= "854" | `DIAGvar' >= "140" & `DIAGvar' <= "208" | `DIAGvar' >= "042" & `DIAGvar' <= "044" | `DIAGvar' >= "781" & `DIAGvar' <= "785"**

**}**

**foreach DIAGvar of varlist DIAG*R {**

**replace OpioidHA_exclusion = 1 if `DIAGvar' >= 202200 & `DIAGvar' <= 202220 | `DIAGvar' >= 202300 & `DIAGvar' <= 202400**

**}**

**/***

**DIAG Exclusions**

**DIAG 801 - 804 Fractures of Skull and Face**

**DIAG 850 - 854 Intracranial Injury, Excluding those with Skull Fracture (includes conscussion)**

**DIAG 140 - 208 Cancer**

**DIAG 042 - 044 (Only 042 is an active code for HIV)**

**DIAG 781 - 785 (Symptoms involving nervous and musculoskeletal systems, Symptoms involving skin and other integumentary tissue, Symptoms concerning nutrition, metabolism, and development, Symptoms involving head and neck, Symptoms involving cardiovascular system)**

**DIAG V220 - V222 (Supervis normal 1st preg, Supervis oth normal preg, Preg state, incidental)**

**DIAG V230 - V240 (Preg w hx of infertility, Preg w hx-trophoblas dis, Preg w hx of abortion, Grand multiparity, Supervision of; pregnancy with other poor obstetric history, Preg w poor reproduct hx, Insufficnt prenatal care, Supervision of; other high-risk pregnancy, Suprv high-risk preg NOS,Postpart care after del)**

***/**

***[CANCER] 2012 - 2015 Cancer**

**replace OpioidHA_exclusion = 1 if CANCER == 1**

***Opioid Heachache Low Value Indicator Variable**

**gen OpioidHA_lowvalue = 0**

**replace OpioidHA_lowvalue = 1 if OpioidHA_inclusion == 1 & OpioidHA_exclusion == 0 & TakingOpioid == 1**

***Imaging Headache Indicator Variable***

**gen HAImaged_lowvalue = 0**

**replace HAImaged_lowvalue = 1 if OpioidHA_inclusion == 1 & OpioidHA_exclusion == 0 & CTorMRI == 1**

***Headache Potential Visit**

**gen HAPotentialVisit = 0**

**replace HAPotentialVisit = 1 if OpioidHA_inclusion == 1 & OpioidHA_exclusion == 0**

**}**

********-------------------------**

***8H. BB for CHF**

***9H. ACE/ARB/ARNI for CHF**

********-------------------------**

**{**

***CHF Inclusion Indicator Variable**

**gen CHF_inclusion = 0**

**foreach DIAGvar of varlist DIAG*3D {**

**replace CHF_inclusion = 1 if `DIAGvar' == "428"**

**}**

**/***

**DIAG Inclusions**

**DIAG 428 Heart Failure**

***/**

***[CHF] 2012 - 2015 Congestive Heart Failure**

**replace CHF_inclusion = 1 if CHF == 1**

***BB CHF Exclusion Indicator Variable**

**gen BBCHF_exclusion = 0**

**foreach RFVvar of varlist RFV* {**

**replace BBCHF_exclusion = 1 if `RFVvar' == 26250 | `RFVvar' == 26200**

**}**

**/***

**RFV Exclusions**

**RFV 26250 Asthma**

**RFV 26200 Emphysema**

***/**

***[ASTHMA] 2012 - 2015 Asthma**

***[COPD] 2012 - 2015 COPD**

**replace BBCAD_exclusion = 1 if ASTHMA == 1**

**replace BBCAD_exclusion = 1 if COPD == 1**

**replace BBCHF_exclusion = 1 if ASTHMA == 1**

**replace BBCHF_exclusion = 1 if COPD == 1**

**foreach DIAGvar of varlist DIAG*3D {**

**replace BBCHF_exclusion = 1 if `DIAGvar' >= "491" & `DIAGvar' <= "493"**

**}**

**foreach DIAGvar of varlist DIAG*R {**

**replace BBCHF_exclusion = 1 if `DIAGvar' >= 142600 & `DIAGvar' <= 142660 | `DIAGvar' == 142781**

**}**

**/***

**DIAG Exclusions**

**DIAG 491 - 493 Chronic Bronchitis, Emphysema, Asthma**

**DIAG 4260 - 4266 Conduction Disorders**

**DIAG 427.81 Sinoatrial node dysfunct**

***/**

***ACE/ARB/ARNI CHF Exclusion Indicator Variable**

**gen ACECHF_exclusion = 0**

**foreach DIAGvar of varlist DIAG*R {**

**replace ACECHF_exclusion = 1 if `DIAGvar' == 127670 | `DIAGvar' == 199510**

**}**

**/***

**DIAG Exclusions**

**DIAG 2767 Hyperpotassemia**

**DIAG 9951 Angioneurotic edema**

***/**

***Exclude Pregnant Patients**

***[PREGNANT] 2012 - 2015 IS PATIENT PREGNANT**

**replace ACECHF_exclusion = 1 if PREGNANT == 1**

***Taking ACE/ARB/ARNI Indicator Variable**

**gen TakingACE = 0**

**foreach RXVar of varlist RX**V2C* {**

**replace TakingACE = 1 if `RXVar' == "042" | `RXVar' == "056"**

**}**

**/*Medications**

**RX Level 2 CAT ID 042 angiotensin converting enzyme inhibitors**

**RX Level 2 CAT ID 056 angiotensin II inhibitors**

***/**

***BB CHF Low Value Indicator Variable**

**gen BBCHF_lowvalue = 0**

**replace BBCHF_lowvalue = 1 if CHF_inclusion == 1 & BBCHF_exclusion == 0 & TakingBB == 0**

***BB CHF High Value Indicator Variable**

**gen BBCHF_Highvalue = 0**

**replace BBCHF_Highvalue = 1 if CHF_inclusion == 1 & BBCHF_exclusion == 0 & TakingBB == 1**

***ACE/ARB/ARNI CHF Low Value Indicator Variable**

**gen ACECHF_lowvalue = 0**

**replace ACECHF_lowvalue = 1 if CHF_inclusion == 1 & ACECHF_exclusion == 0 & TakingACE == 0**

***ACE/ARB/ARNI CHF High Value Indicator Variable**

**gen ACECHF_Highvalue = 0**

**replace ACECHF_Highvalue = 1 if CHF_inclusion == 1 & ACECHF_exclusion == 0 & TakingACE == 1**

***CHF Potential Visit Indicator Variable***

**gen BBCHFPotentialVisit = 0**

**replace BBCHFPotentialVisit = 1 if CHF_inclusion == 1 & BBCHF_exclusion == 0**

**gen ACECHFPotentialVisit = 0**

**replace ACECHFPotentialVisit = 1 if CHF_inclusion == 1 & ACECHF_exclusion == 0**

**}**

********-------------------------**

***10H. Osteoporosis Tx**

********-------------------------**

**{**

***Osteoporosis Inclusion Indicator Variable**

**gen Osteoporosis_inclusion = 0**

**foreach DIAGvar of varlist DIAG*3D {**

**replace Osteoporosis_inclusion = 1 if `DIAGvar' == "733"**

**}**

**/***

**DIAG Inclusions**

**DIAG 7330 (Change to 3 Digit 733 Osteoporosis?)**

***/**

***[OSTPRSIS] 2012 - 2015 Osteoporosis**

**replace Osteoporosis_inclusion = 1 if OSTPRSIS == 1**

***Osteoporosis Exclusion Indicator Variable**

**gen Osteoporosis_exclusion = 0**

**foreach DIAGvar of varlist DIAG*R {**

**replace Osteoporosis_exclusion = 1 if `DIAGvar' == 173345**

**}**

**/***

**DIAG Exclusions**

**DIAG 733.45 Aseptic Necrosis of Jaw**

***/**

***Osteoporosis Tx Indicator Variable**

**gen OsteoporosisTx = 0**

**foreach RXVar of varlist RX**V2C* {**

**replace OsteoporosisTx = 1 if `RXVar' == "411" | `RXVar' == "417" | `RXVar' == "418" | `RXVar' == "409"**

**}**

**foreach RXVar of varlist RX**V3C* {**

**replace OsteoporosisTx = 1 if `RXVar' == "217" | `RXVar' == "415"**

**}**

**/***

**Medications**

**RX Level 3 CAT ID 217 bisphosphonates**

**RX Level 3 CAT ID 415 miscellaneous bone resorption inhibitors**

**RX Level 2 CAT ID 418 parathyroid hormone and analogs**

**RX Level 2 CAT ID 417 selective estrogen receptor modulators**

**RX Level 2 CAT ID 411 calcitonin**

**RX Level 2 CAT ID 409 Bone Resporption Inhibitors**

***/**

***Osteoporosis Tx Low Value Indicator Variable***

**gen OsteoporosisTx_lowvalue = 0**

**replace OsteoporosisTx_lowvalue = 1 if Osteoporosis_inclusion == 1 & Osteoporosis_exclusion == 0 & OsteoporosisTx == 0**

***Osteoporosis Tx High Value Indicator Variable***

**gen OsteoporosisTx_highvalue = 0**

**replace OsteoporosisTx_highvalue = 1 if Osteoporosis_inclusion == 1 & Osteoporosis_exclusion == 0 & OsteoporosisTx == 1**

***Osteoporosis Potential Visit Indicator Variable***

**gen OsteoporosisPotentialVisit = 0**

**replace OsteoporosisPotentialVisit = 1 if Osteoporosis_inclusion == 1 & Osteoporosis_exclusion == 0**

**}**

********-------------------------**

***5L. Abx for URI**

********-------------------------**

**{**

***URI Inclusion Indicator Variable**

**gen URI_inclusion = 0**

**foreach RFVvar of varlist RFV* {**

**replace URI_inclusion = 1 if `RFVvar' == 26100 | `RFVvar' == 26000 | `RFVvar' == 14450 | `RFVvar' == 14750 | `RFVvar' == 14551**

**}**

**/***

**RFV Inclusions**

**RFV 26100 Bronchitis**

**RFV 26000 Upper Respiratory Infections Except Tonsillitis**

**RFV 14450 Head cold, upper respiratory infection (coryza)**

**RFV 14750 congestion in Chest**

**RFV 14551 Soreness of Throat**

***/**

**foreach DIAGvar of varlist DIAG*3D {**

**replace URI_inclusion = 1 if `DIAGvar' == "460" | `DIAGvar' == "462" | `DIAGvar' == "464" | `DIAGvar' == "465" | `DIAGvar' == "466" | `DIAGvar' == "490"**

**}**

**foreach DIAGvar of varlist DIAG*R {**

**replace URI_inclusion = 1 if `DIAGvar' == 149121**

**}**

**/***

**DIAG Inclusions**

**DIAG 460 Acute Nasopharyngitis**

**DIAG 462 Acute Pharyngitis**

**DIAG 464 Acute laryngitis and tracheitis**

**DIAG 465 Acute upper respiratory infections of multiple or unspecified sites**

**DIAG 466 Acute bronchitis and bronchiolitis**

**DIAG 490 Bronchitis NOS**

**DIAG 49121 Chronic obstructive asthma with status asthmaticus**

***/**

***URI and Major Reason for this Visit is a New Problem Indicator Variable***

**gen NewURI_inclusion = 0**

**replace NewURI_inclusion = 1 if URI_inclusion == 1 & MAJOR == 1**

***URI Exclusion Indicator Variable**

**gen URI_exclusion = 0**

**foreach DIAGvar of varlist DIAG*3D {**

**replace URI_exclusion = 1 if `DIAGvar' == "590" | `DIAGvar' == "597" | `DIAGvar' == "421" | `DIAGvar' == "522" | `DIAGvar' == "523" | `DIAGvar' == "730" | `DIAGvar' == "008" | `DIAGvar' == "009" | `DIAGvar' == "026" | `DIAGvar' == "031" | `DIAGvar' == "035" | `DIAGvar' == "038" | `DIAGvar' == "041" | `DIAGvar' == "083" | `DIAGvar' == "088" | `DIAGvar' == "130" | `DIAGvar' == "131" | `DIAGvar' == "250" | `DIAGvar' == "707" | `DIAGvar' == "614" | `DIAGvar' == "615" | `DIAGvar' == "616" | `DIAGvar' == "675" | `DIAGvar' == "601" | `DIAGvar' == "604" | `DIAGvar' == "748" | `DIAGvar' == "540" | `DIAGvar' == "541" | `DIAGvar' == "562" | `DIAGvar' == "566" | `DIAGvar' == "567" | `DIAGvar' >= "574" & `DIAGvar' <= "577" | `DIAGvar' == "042" | `DIAGvar' == "V08" | `DIAGvar' == "V42" | `DIAGvar' >= "031" & `DIAGvar' <= "040" | `DIAGvar' >= "320" & `DIAGvar' <= "324" | `DIAGvar' >= "680" & `DIAGvar' <= "686" | `DIAGvar' >= "090" & `DIAGvar' <= "099" | `DIAGvar' >= "870" & `DIAGvar' <= "897" | `DIAGvar' == "V42" | `DIAGvar' >= "140" & `DIAGvar' <= "208"**

**}**

**foreach DIAGvar of varlist DIAG*R {**

**replace URI_exclusion = 1 if `DIAGvar' == 159500 | `DIAGvar' == 159590 | `DIAGvar' == 179070 | `DIAGvar' == 100620 | `DIAGvar' == 107988 | `DIAGvar' == 107998 | `DIAGvar' == 137601 | `DIAGvar' == 137602 | `DIAGvar' >= 138010 & `DIAGvar' <= 138016 | `DIAGvar' == 138022 | `DIAGvar' == 138023 | `DIAGvar' == 152720 | `DIAGvar' == 152730 | `DIAGvar' == 152830 | `DIAGvar' >= 171100 & `DIAGvar' <= 171109 | `DIAGvar' >= 171140 & `DIAGvar' <= 171149 | `DIAGvar' >= 171180 & `DIAGvar' <= 171189 | `DIAGvar' >= 171190 & `DIAGvar' <= 171199 | `DIAGvar' == 172800 | `DIAGvar' == 199931 | `DIAGvar' == 170610 | `DIAGvar' == 161600 | `DIAGvar' == 161610 | `DIAGvar' == 161630 | `DIAGvar' == 161640 | `DIAGvar' == 161690 | `DIAGvar' == 128930 | `DIAGvar' == 137313 | `DIAGvar' == 128930 | `DIAGvar' == 137313 | `DIAGvar' == 170480 | `DIAGvar' == 170583 | `DIAGvar' == 191170 | `DIAGvar' == 191410 | `DIAGvar' == 191430 | `DIAGvar' == 191450 | `DIAGvar' == 191470 | `DIAGvar' == 191490 | `DIAGvar' == 191510 | `DIAGvar' == 191530 | `DIAGvar' == 191550 | `DIAGvar' == 191570 | `DIAGvar' == 191590 | `DIAGvar' == 191950 | `DIAGvar' == 193310 | `DIAGvar' == 200160 | `DIAGvar' == 200189 | `DIAGvar' == 200190 | `DIAGvar' == 164660 | `DIAGvar' >= 164790 & `DIAGvar' <= 164794 | `DIAGvar' >= 165840 & `DIAGvar' <= 165843 | `DIAGvar' == 160310 | `DIAGvar' == 151911 | `DIAGvar' == 151980 | `DIAGvar' == 171481 | `DIAGvar' == 177020 | `DIAGvar' == 128920 | `DIAGvar' == 156971 | `DIAGvar' == 157200 | `DIAGvar' == 107953 | `DIAGvar' == 179571 | `DIAGvar' == 199851 | `DIAGvar' == 199859 | `DIAGvar' >= 199660 & `DIAGvar' <= 199669 | `DIAGvar' >= 199680 & `DIAGvar' <= 199689 | `DIAGvar' >= 164660 & `DIAGvar' <= 164664**

**}**

**/***

**DIAG Exclusions**

**DIAG 590 Infections of Kideny**

**DIAG 597 Urethritis, not sexually transmitted, and urethral syndrome**

**DIAG 5950 Acute cystitis**

**DIAG 5959 Cystitis NOS**

**DIAG 5990 Urinary tract dis NOS**

**DIAG 421 Acute and subacute endocarditis**

**DIAG 522 Diseases of pulp and periapical tissues**

**DIAG 523 Gingival and periodontal diseases**

**DIAG 730 Osteomyelitis, periostitis, and other infections involving bone**

**DIAG 7907 Bacteremia**

**DIAG 0062 Amebic nondysent colitis**

**DIAG 00843 (All 008 Intestinal infections due to other organisms)**

**DIAG 00845 (All 008 Intestinal infections due to other organisms)**

**DIAG 00849 (All 008 Intestinal infections due to other organisms)**

**DIAG 0090 (All 009 Ill-defined intestinal infections?)**

**DIAG 0092 (All 009 Ill-defined intestinal infections?)**

**DIAG 0093 (All 009 Ill-defined intestinal infections?)**

**DIAG 0261 (Change to 3 digit 026 Rat-bite fever)**

**DIAG 0310 (Change to 3 digit 031 Diseases due to other mycobacteria)**

**DIAG 0318 (Change to 3 digit 031 Diseases due to other mycobacteria)**

**DIAG 0319 (Change to 3 digit 031 Diseases due to other mycobacteria)**

**DIAG 035 Erysipelas**

**DIAG 0380 (Change to 3 digit 038 Septicemia)**

**DIAG 03810 (Change to 3 digit 038 Septicemia)**

**DIAG 0384 (Change to 3 digit 038 Septicemia)**

**DIAG 04186 (Change to 3 digit 041 Bacterial infection in conditions classified elsewhere and of unspecified site)**

**DIAG 07988 Oth spcf chlamydial infc**

**DIAG 07998 Chlamydial infection NOS**

**DIAG 0830 (Change to 3 digit 083 Other rickettsioses)**

**DIAG 08881 (Change to 3 digit 088 Other arthropod-borne diseases)**

**DIAG 1300 (Change to 3 digit 130 Toxoplasmosis)**

**DIAG 37601 Orbital cellulitis (Added this)**

**DIAG 37602 Orbital periostitis**

**DIAG 38011 (Change to 38010 - 38016 Infective otitis externa)**

**DIAG 38022 Acute otitis externa NEC (Added this)**

**DIAG 38023 Chr otitis externa NEC**

**DIAG 5272 Sialoadenitis**

**DIAG 5273 Salivary gland abscess (Added this)**

**DIAG 5283 Cellulitis/abscess mouth**

**DIAG 71101 (Change to 71100 - 71109 Pyogenic arthritis)**

**DIAG 71103 (Change to 71100 - 71109 Pyogenic arthritis)**

**DIAG 71108 (Change to 71100 - 71109 Pyogenic arthritis)**

**DIAG 71140 - 71149 Arthropathy associated with other bacterial diseases (Added this)**

**DIAG 71184 (Change to 71180 - 71189 Arthropathy associated with other infectious and parasitic diseases)**

**DIAG 71186 (Change to 71180 - 71189 Arthropathy associated with other infectious and parasitic diseases)**

**DIAG 71189 (Change to 71180 - 71189 Arthropathy associated with other infectious and parasitic diseases)**

**DIAG 71195 (Change to 71190 - 71199 Unspecified infective arthritis)**

**DIAG 71197 (Change to 71190 - 71199 Unspecified infective arthritis)**

**DIAG 71198 (Change to 71190 - 71199 Unspecified infective arthritis)**

**DIAG 7280 Infective myositis**

**DIAG 99931 (Change to 99931 - 99939 Other infection; as complication of medical care;)**

**DIAG 7061 Acne NEC**

**DIAG 6160 Cervicitis**

**DIAG 61610 - 61611 Vaginitis and vulvovaginitis**

**DIAG 6163 Bartholin's glnd abscess**

**DIAG 6164 Abscess of vulva NEC**

**DIAG 6169 Female gen inflam NOS**

**DIAG 13101 (Change to 3 digit 131 Trichomoniasis)**

**DIAG 2893 Lymphadenitis NOS**

**DIAG 37313 Abscess of eyelid**

**DIAG 7048 Hair diseases NEC (Do we want this?)**

**DIAG 70583 Hidradenitis**

**DIAG 9117 Foreign body trunk-infec**

**DIAG 9141 Abrasion hand-infected**

**DIAG 9143 Blister hand-infected (Added this)**

**DIAG 9145 Insect bite hand-infect (Added this)**

**DIAG 9147 Foreign body hand-infect (Added this)**

**DIAG 9149 Superf inj hand NEC-inf (Added this)**

**DIAG 9151 Abrasion finger-infected**

**DIAG 9153 Blister finger-infected (Added this)**

**DIAG 9155 Insect bite finger-infec (Added this)**

**DIAG 9157 Foreign body finger-inf (Added this)**

**DIAG 9159 Suprf inj finger NEC-inf**

**DIAG 9195 Insect bite NEC-infected**

**DIAG 9331 Foreign body in larynx**

**DIAG E9060 Dog bite (Cannot find a way to code this)**

**DIAG E9063 Foreign body in larynx (Cannot find a way to code this)**

**DIAG 250 Diabetes mellitus**

**DIAG V016 Venereal dis contact**

**DIAG V0189 Contact with or exposure to other communicable diseases**

**DIAG V019 Communic dis contact NOS**

**DIAG 707 Chronic ulcer of skin**

**DIAG 614 Inflammatory disease of ovary, fallopian tube, pelvic cellular tissue, and peritoneum**

**DIAG 615 Inflammatory diseases of uterus, except cervix**

**DIAG 616 Inflammatory disease of cervix, vagina, and vulva**

**DIAG 6466 Infections of genitourinary tract in pregnancy**

**DIAG 64790 - 64794 Unspecified infection or infestation, complicating pregnancy, childbirth, or the puerperium;**

**DIAG 65840 - 65843 Infection of amniotic cavity**

**DIAG 6751 (Change to 3 digit 675 Infections of the breast and nipple associated with childbirth)**

**DIAG 67510 (Change to 3 digit 675 Infections of the breast and nipple associated with childbirth)**

**DIAG 67514 (Change to 3 digit 675 Infections of the breast and nipple associated with childbirth)**

**DIAG 6759 (Change to 3 digit 675 Infections of the breast and nipple associated with childbirth)**

**DIAG 67590 (Change to 3 digit 675 Infections of the breast and nipple associated with childbirth)**

**DIAG 67591 (Change to 3 digit 675 Infections of the breast and nipple associated with childbirth)**

**DIAG 601 Inflammatory diseases of prostate**

**DIAG 604 Orchitis and epididymitis**

**DIAG 6031 Infected hydrocele**

**DIAG 51911 Acute bronchospasm**

**DIAG 5198 Resp system disease NEC**

**DIAG 7484 (Change to 3 digit 748 Congenital anomalies of respiratory system)**

**DIAG 7485 (Change to 3 digit 748 Congenital anomalies of respiratory system)**

**DIAG 74861 (Change to 3 digit 748 Congenital anomalies of respiratory system)**

**DIAG 71481 Rheumatoid lung**

**DIAG 7702 NB interstit emphysema**

**DIAG 540 Acute appendicitis**

**DIAG 541 Appendicitis NOS**

**DIAG 562 Diverticula of intestine**

**DIAG 566 Anal & rectal abscess**

**DIAG 567 Peritonitis and retroperitoneal infections**

**DIAG 574 - 577 Cholelithiasis, Other disorders of gallbladder, Other disorders of biliary tract, Diseases of pancreas**

**DIAG 2892 Mesenteric lymphadenitis**

**DIAG 56971 Pouchitis**

**DIAG 5720 Abscess of liver**

**DIAG 042 Human immunodeficiency virus [HIV] disease**

**DIAG V08 Asymp hiv infectn status**

**DIAG 07953 Hiv-2 infection oth dis**

**DIAG 79571 Nonspcf serlgc evdnc hiv**

**DIAG 99851 Infected postop seroma**

**DIAG 99859 Other postop infection**

**DIAG 99660 - 99669 Infection & inflammatory reaction due to internal prosthetic device, implant, & graft**

**DIAG V42 Organ or tissue replaced by transplant**

**DIAG E8780 Abn react-surg proc NEC (Cannot find a way to code this)**

**DIAG 99680 - 99689 Complications of transplanted organ**

**DIAG 031 - 040 Diseases due to other mycobacteria, Diphtheria, Whooping cough, Streptococcal sore throat and scarlet fever, Erysipelas, Meningococcal infection, Tetanus, Septicemia, Actinomycotic infections, Other bacterial diseases**

**DIAG 320 - 324 Bacterial meningitis, Meningitis due to other organisms, Meningitis of unspecified cause, 323 Encephalitis, myelitis, and encephalomyelitis, Intracranial and intraspinal abscess**

**DIAG 680 - 686 INFECTIONS OF SKIN AND SUBCUTANEOUS TISSUE**

**DIAG 090 - 099 SYPHILIS AND OTHER VENEREAL DISEASES**

**DIAG 870 - 897 OPEN WOUND**

**DIAG 64660 - 64664 Infections of genitourinary tract in pregnancy**

**DIAG V420 - V429 (Change to 3 digit V42 Organ or tissue replaced by transplant)**

**DIAG 140 - 208 Cancer**

***/**

***[COPD] 2012 - 2015 Chronic obstructive pulmonary disease (COPD)**

***[CANCER] 2012 - 2015 Cancer**

***[ERADMHOS] 2012 - 2015 Refer to emergency department/Admit to hospital**

***[HIV] 2014 - 2015 HIV**

**replace URI_exclusion = 1 if COPD == 1**

**replace URI_exclusion = 1 if CANCER == 1**

**replace URI_exclusion = 1 if ERADMHOS == 1**

**replace URI_exclusion = 1 if HIV == 1**

***Taking Antibiotic Indicator Variable**

**gen TakingAntibiotic = 0**

**foreach RXVar of varlist RX**V2C* {**

**replace TakingAntibiotic = 1 if `RXVar' >= "008" & `RXVar' <= "018" | `RXVar' == "240" | `RXVar' == "315" | `RXVar' == "406"**

**}**

**/***

**Medications**

**RX Level 2 CAT ID 008 carbapenems**

**RX Level 2 CAT ID 009 cephalosporins**

**RX Level 2 CAT ID 010 leprostatics**

**RX Level 2 CAT ID 011 macrolide derivatives**

**RX Level 2 CAT ID 012 miscellaneous antibiotics**

**RX Level 2 CAT ID 013 penicillins**

**RX Level 2 CAT ID 014 quinolones**

**RX Level 2 CAT ID 015 sulfonamides**

**RX Level 2 CAT ID 016 tetracyclines**

**RX Level 2 CAT ID 017 urinary anti-infectives**

**RX Level 2 CAT ID 018 aminoglycosides**

**RX Level 2 CAT ID 240 lincomycin derivatives**

**RX Level 2 CAT ID 315 glycylcyclines**

**RX Level 2 CAT ID 406 glycopeptide antibiotics**

***/**

**/***

**foreach MEDVar of varlist MED** {**

**replace TakingAntibiotic = 1 if `MEDVar' ==**

**}**

**/*Medications**

**MED1 - MED10**

***/**

**foreach DRUGIDVar of varlist DRUGID** {**

**replace TakingAntibiotic = 1 if `DRUGIDVar' ==**

**}**

**/*Drug ID**

***/**

***/**

***Abx for URI Low Value Indicator Variable**

**gen AbxURI_lowvalue = 0**

**replace AbxURI_lowvalue = 1 if NewURI_inclusion == 1 & URI_exclusion == 0 & TakingAntibiotic == 1**

***Abx for URI Potential Visits**

**gen AbxURIPotentialVisit = 0**

**replace AbxURIPotentialVisit = 1 if NewURI_inclusion == 1 & URI_exclusion == 0**

**}**

********-------------------------**

***6L. GME ECG**

***7L. GME UA**

********-------------------------**

**{**

***GME Inclusion Indicator Variable**

**gen GME_inclusion = 0**

**foreach RFVvar of varlist RFV* {**

**replace GME_inclusion = 1 if `RFVvar' == 31000**

**}**

**/***

**RFV Inclusions**

**RFV 31000 General medical examination**

***/**

**foreach DIAGvar of varlist DIAG*R {**

**replace GME_inclusion = 1 if `DIAGvar' == 207000 | `DIAGvar' == 207090**

**}**

**/***

**DIAG Inclusions**

**DIAG V700 Routine medical exam**

**DIAG V709 General medical exam NOS**

***/**

***ECG Exclusion Indicator Variable**

**gen ECG_exclusion = 0**

**foreach RFVvar of varlist RFV* {**

**replace ECG_exclusion = 1 if `RFVvar' == 10500 | `RFVvar' == 10501 | `RFVvar' == 10502 | `RFVvar' == 10503 | `RFVvar' == 10300 | `RFVvar' == 10200 | `RFVvar' == 10350 | `RFVvar' == 10351 | `RFVvar' == 12600 | `RFVvar' == 12601 | `RFVvar' == 12602 | `RFVvar' == 12603 | `RFVvar' == 12650 | `RFVvar' == 12700 | `RFVvar' == 14150 | `RFVvar' == 14200 | `RFVvar' == 14250 | `RFVvar' == 14300 | `RFVvar' == 14301 | `RFVvar' == 14302 | `RFVvar' == 14552 | `RFVvar' == 15250 | `RFVvar' == 58400 | `RFVvar' == 25000 | `RFVvar' == 25050 | `RFVvar' == 25100 | `RFVvar' == 25150 | `RFVvar' == 25200 | `RFVvar' == 25250 | `RFVvar' == 25300**

**}**

**/***

**RFV Exclusions**

**rfv 1050.0 Chest pain and related symptoms (not referable to a specific body system)**

**rfv 1050.1 Chest pain**

**rfv 1050.2 Chest discomfort, pressure, tightness, and heaviness, including chest pressure**

**rfv 1050.3 Burning sensation in the chest**

**rfv 1030.0 Fainting (syncope), including blacking out, fainting spells, and passing out**

**rfv 1020.0 General weakness**

**rfv 1035.0 Symptoms of fluid abnormalities, including fluid retention and holding water**

**rfv 1035.1 Edema, including ankle swelling, bloated dropsy legs, and peripheral swollen with water**

**rfv 1260.0 Abnormal pulsations and palpitations**

**rfv 1260.1 Increased heartbeat, including pulse too fast and rapid heartbeat**

**rfv 1260.2 Decreased heartbeat, including pulse too slow and slow heart**

**rfv 1260.3 Irregular heartbeat, including fluttering, jumping, racing, and skipped beat**

**rfv 1265.0 Heart pain, including anginal pain, heart distress, and pain over heart**

**rfv 1270.0 Other symptoms of the heart, including bad heart condition, poor heart, and weak heart**

**rfv 1415.0 Shortness of breath, including breathlessness and out-of-breath sensation of suffocation**

**rfv 1420.0 Labored or difficult breathing (dyspnea), including anoxia (cannot breathe), hypoxia, smothering respiratory distress, and trouble breathing**

**rfv 1425.0 Wheezing, including sighing respiration**

**rfv 1430.0 Breathing problems, including hurts to breathe**

**rfv 1430.1 Disorders of respiratory sound, including abnormal breathing sounds, snoring, rales, rattles, and stridor**

**rfv 1430.2 Rapid breathing (hyperventilation)**

**rfv 1455.2 Throat pain**

**rfv 1525.0 Nausea, including feel like throwing up, nervous stomach, sick to stomach, and upset stomach**

**rfv 5840.0 Unconsciousness**

**rfv 2500.0 Rheumatic fever and chronic rheumatic heart disease, including chorea**

**rfv 2505.0 Hypertension with involvement of target organs, including hypertensive cardiovascular disease, hypertensive heart disease, pulmonary hypertension, and renal hypertension**

**rfv 2510.0 Hypertension and hypertensive, including high blood pressure**

**rfv 2515.0 Ischemic heart disease, including angina pectoris, arteriorsclerotic cardiovascular disease, arteriosclerotic heart disease, coronary artery disease, heart attack, and myocardial infarction**

**rfv 2520.0 Other heart disease, including aortic valve stenosis, arrhythmia NOS, atrial fibrillation, cardiac arrhythmia, cardiac dysrhythmia, cardiomyopathy, congestive cardiomyopathy, congestive heart failure, cor pulmonale, heart failure, heart murmur, mitral valve prolapse, mitral valve regurgitation, paroxysmal tachycardia, premature ventricular contractions, and ventricular tachycardia**

**rfv 2525.0 Cerebrovascular disease, including carotid stenosis, cerebral arteriosclerosis, cerebral hemorrhage, cerebral stenosis, cerebrovascular accident, stroke, and transient ischemic attack**

**rfv 2530.0 Atherosclerosis, including arteriosclerosis and hardening of ther arteries (excluding cerebral arteriosclerosis)**

***/**

**foreach DIAGvar of varlist DIAG*3D {**

**replace ECG_exclusion = 1 if `DIAGvar' >= "420" & `DIAGvar' <= "429" | `DIAGvar' == "785" | `DIAGvar' == "786" | `DIAGvar' >= "584" & `DIAGvar' <= "586" | `DIAGvar' >= "401" & `DIAGvar' <= "405" | `DIAGvar' >= "430" & `DIAGvar' <= "438" | `DIAGvar' >= "410" & `DIAGvar' <= "414"**

**}**

**foreach DIAGvar of varlist DIAG*R {**

**replace ECG_exclusion = 1 if `DIAGvar' == 178020 | `DIAGvar' == 195911**

**}**

**/***

**DIAG Exclusions**

**DIAG 426 (Think we should do 420 - 429 instead, Other forms of Heart Disease)**

**DIAG 428 (Think we should do 420 - 429 instead, Other forms of Heart Disease)**

**DIAG 427 (Think we should do 420 - 429 instead, Other forms of Heart Disease)**

**DIAG 7802 Syncope and collapse**

**DIAG 7851 (Change to 3 digit 785 Symptoms involving cardiovascular system)**

**DIAG 7860 (Change to 3 digit 786 Symptoms involving respiratory system and other chest symptoms)**

**DIAG 7863 (Change to 3 digit 786 Symptoms involving respiratory system and other chest symptoms)**

**DIAG 7865 (Change to 3 digit 786 Symptoms involving respiratory system and other chest symptoms)**

**DIAG 95911 Injury of chest wall NEC**

**DIAG 584 - 586 Acute Kideny Failure, CKD, Renal failure NOS**

**DIAG 401 - 405 Hypertensive Disease**

**DIAG 430 - 437 (Extend to 438 from 437 Cerebrovascular Disease)**

**DIAG 410 - 414 Ischemic Heart Disease**

***/**

***[CAD] 2014 - 2015 Coronary artery disease (CAD), ischemic heart disease (IHD), or history of myocardial infarction (MI)**

***[IHD] 2012 - 2015 Includes angina pectoris, coronary atherosclerosis, acute myocardial infarction, and other forms of ischemic heart disease.**

***[CHF] 2012 - 2015 Congestive Heart Failure**

***[HTN] 2012 - 2015 Hypertension**

***[CEBVD] 2012 - 2105 Cerebrovascular disease/History of stroke (CVA) or transient ischemic attack (TIA)**

***[CRF] 2012 - 2013 Chronic Renal Failure**

***[ESRD] 2013 - 2015 End stage renal disease (ESRD)**

**replace ECG_exclusion = 1 if CAD == 1**

**replace ECG_exclusion = 1 if IHD == 1**

**replace ECG_exclusion = 1 if CHF == 1**

**replace ECG_exclusion = 1 if HTN == 1**

**replace ECG_exclusion = 1 if CEBVD == 1**

**replace ECG_exclusion = 1 if CRF == 1**

**replace ECG_exclusion = 1 if ESRD == 1**

***UA Exclusion Indicator Variable**

**gen UA_exclusion = 0**

**foreach RFVvar of varlist RFV* {**

**replace UA_exclusion = 1 if `RFVvar' == 16652 | `RFVvar' == 16702 | `RFVvar' == 16800 | `RFVvar' == 16750 | `RFVvar' == 17102 | `RFVvar' == 27000 | `RFVvar' == 27050 | `RFVvar' == 27100 | `RFVvar' == 17753 | `RFVvar' == 27200 | `RFVvar' == 32050 | `RFVvar' == 16400 | `RFVvar' == 62000 | `RFVvar' == 16401 | `RFVvar' == 16402 | `RFVvar' == 16403 | `RFVvar' == 16450 | `RFVvar' == 16451 | `RFVvar' == 16500**

**}**

**/***

**RFV Exclusions**

**rfv 1665.2 Bladder infection**

**rfv 1670.2 Kidney infection, NOS**

**rfv 1680.0 Passed stones**

**rfv 1675.0 Urinary tract infection**

**rfv 1710.2 Prostate infection**

**rfv 2700.0 Cystitis**

**rfv 2705.0 Urinary tract disease (except cystitis), including bladder stones, glomerulonephritis, glomerulonephrosis, kidney stones, neurogenic bladder, pyelonephritis, renal failure, ureteral calculus, and urethritis**

**rfv 2710.0 Disease of the male genital organs, including benign prostatic hypertrophy, epididymitis, hydrovele, Peyronie disease, phimosis, and prostatitis**

**rfv 1775.3 Pelvic infection, NOS**

**rfv 2720.0 Pelvic inflammatory disease, including oophoritic, pelvic peritonitis, and salpingitis**

**rfv 3205.0 Prenatal examination, routine, including normal antepartum visit, pregnancy NOS, and routine obstetrical care**

**rfv 1640.0 Abnormalities of urine**

**rfv 6200.0 Abnormal findings of urine tests**

**rfv 1640.1 Blood in urine (hematuria)**

**rfv 1640.2 Pus in urine**

**rfv 1640.3 Unusual color or odor**

**rfv 1645.0 Frequency and urgency of urination**

**rfv 1645.1 Excessive urination, night (nocturia)**

**rfv 1650.0 Painful urination, including burning and discomfort**

***/**

**foreach DIAGvar of varlist DIAG*3D {**

**replace UA_exclusion = 1 if `DIAGvar' >= "580" & `DIAGvar' <= "608" | `DIAGvar' == "788" | `DIAGvar' == "V22" | `DIAGvar' == "V23"**

**}**

**/***

**DIAG Exclusions**

**DIAG V22 Normal Pregnancy**

**DIAG V23 Supervision of high-risk pregnancy**

**DIAG 580 - 608 NEPHRITIS, NEPHROTIC SYNDROME, AND NEPHROSIS (580-589), OTHER DISEASES OF THE URINARY SYSTEM (590-599), DISEASES OF MALE GENITAL ORGANS (600-608)**

**DIAG 7880 - 7882 (Change to 3 digit 788 Symptoms involving the urinary system - will include all previous codes and incontinence)**

**DIAG 7884 - 7887 (Change to 3 digit 788 Symptoms involving the urinary system - will include all previous codes and incontinence)**

***/**

***[CRF] 2012 - 2013 Chronic Renal Failure**

***[ESRD] 2013 - 2015 End stage renal disease (ESRD)**

***[CKD] 2014 - 2015 Chornic Kidney Disease (CKD)**

**replace UA_exclusion = 1 if CRF == 1**

**replace UA_exclusion = 1 if ESRD == 1**

**replace UA_exclusion = 1 if CKD == 1**

***GME ECG Low Value Indicator Variable***

**gen GMEECG_lowvalue = 0**

**replace GMEECG_lowvalue = 1 if GME_inclusion == 1 & ECG_exclusion == 0 & EKG == 1**

***GME UA Low Value Indicator Variable***

**gen GMEUA_lowvalue = 0**

**replace GMEUA_lowvalue = 1 if GME_inclusion == 1 & UA_exclusion == 0 & URINE == 1**

***GME Potential Visit Indicator Variable***

**gen GMEECGPotentialVisit = 0**

**replace GMEECGPotentialVisit = 1 if GME_inclusion == 1 & ECG_exclusion == 0**

**gen GMEUAPotentialVisit = 0**

**replace GMEUAPotentialVisit = 1 if GME_inclusion == 1 & UA_exclusion == 0**

**}**

**}**

***-------------------------------------------------**

***Generate Low/High Value Vists/Counts Indicators**

***-------------------------------------------------**

***Generate Low Value and High Value Visit Indicator**

**gen LowValueVisit = 0**

**replace LowValueVisit = 1 if BackPainImaging_LowValue == 1 | BackPainOpioid_LowValue == 1 | OpioidHA_lowvalue == 1 | HAImaged_lowvalue == 1 | AbxURI_lowvalue == 1 | GMEECG_lowvalue == 1 | GMEUA_lowvalue == 1**

**gen HighValueVisit = 0**

**replace HighValueVisit = 1 if ASACAD_highvalue == 1 | BBCAD_Highvalue == 1 | StatinCAD_Highvalue == 1 | ACAfib_Highvalue == 1 | StatinDM_Highvalue == 1 | AntiPltCVD_Highvalue == 1 | DepTx_highvalue == 1 | BBCHF_Highvalue == 1 | ACECHF_Highvalue == 1 | OsteoporosisTx_highvalue == 1**

***Generate Low Value and High Value Service Count Variable**

**egen LowValueServiceCount = rowtotal(BackPainImaging_LowValue BackPainOpioid_LowValue OpioidHA_lowvalue HAImaged_lowvalue AbxURI_lowvalue GMEECG_lowvalue GMEUA_lowvalue)**

**egen HighValueServiceCount = rowtotal(ASACAD_highvalue BBCAD_Highvalue StatinCAD_Highvalue ACAfib_Highvalue StatinDM_Highvalue AntiPltCVD_Highvalue DepTx_highvalue BBCHF_Highvalue ACECHF_Highvalue OsteoporosisTx_highvalue)**

***Generate Low Value Potential Visit Indicator Variable (Visit for LBP, HA, URI, GME)**

**gen LowValuePotential = 0**

**replace LowValuePotential = 1 if BackPainPotentialVisit == 1 | HAPotentialVisit == 1 | AbxURIPotentialVisit == 1 | GMEUAPotentialVisit == 1 | GMEECGPotentialVisit == 1**

***Generate Low Value Potential Visit Subpopulation Indicator Variables**

**gen PreAdults_LowValuePotential = 0**

**replace PreAdults_LowValuePotential = 1 if PreExpansion_Adults_13States == 1 & LowValuePotential == 1**

**gen PreMcaid_LowValuePotential = 0**

**replace PreMcaid_LowValuePotential = 1 if PreExpan_McaidAdults_13States == 1 & LowValuePotential == 1**

**gen PreNewMcaid_LowValuePotential = 0**

**replace PreNewMcaid_LowValuePotential = 1 if PreExAccNewMcaidAdults_13States == 1 & LowValuePotential == 1**

**gen Adults_LowValuePotential = 0**

**replace Adults_LowValuePotential = 1 if Adults_13States == 1 & LowValuePotential == 1**

**gen Mcaid_LowValuePotential = 0**

**replace Mcaid_LowValuePotential = 1 if McaidAdults_13States == 1 & LowValuePotential == 1**

**gen NewMcaid_LowValuePotential = 0**

**replace NewMcaid_LowValuePotential = 1 if AcceptNewMcaidAdults_13States == 1 & LowValuePotential == 1**

***Generate High Value Potential Visit Indicator Variable**

**gen HighValuePotential = 0**

**replace HighValuePotential = 1 if ASACADPotentialVisit == 1 | BBCADHighValuePotential == 1 | StatinCADHighValuePotential == 1 | ACAfibPotentialVisit == 1 | StatinDMPotentialVisit == 1 | AntiPltCVDPotentialVisit == 1 | Dep_inclusion == 1 | BBCHFPotentialVisit == 1 | ACECHFPotentialVisit == 1 | OsteoporosisPotentialVisit == 1**

***Generate High Value Potential Visit Subpopulation Indicator Variables**

**gen PreAdults_HighValuePotential = 0**

**replace PreAdults_HighValuePotential = 1 if PreExpansion_Adults_13States == 1 & HighValuePotential == 1**

**gen PreMcaid_HighValuePotential = 0**

**replace PreMcaid_HighValuePotential = 1 if PreExpan_McaidAdults_13States == 1 & HighValuePotential == 1**

**gen PreNewMcaid_HighValuePotential = 0**

**replace PreNewMcaid_HighValuePotential = 1 if PreExAccNewMcaidAdults_13States == 1 & HighValuePotential == 1**

**gen Adults_HighValuePotential = 0**

**replace Adults_HighValuePotential = 1 if Adults_13States == 1 & HighValuePotential == 1**

**gen Mcaid_HighValuePotential = 0**

**replace Mcaid_HighValuePotential = 1 if McaidAdults_13States == 1 & HighValuePotential == 1**

**gen NewMcaid_HighValuePotential = 0**

**replace NewMcaid_HighValuePotential = 1 if AcceptNewMcaidAdults_13States == 1 & HighValuePotential == 1**

***-------------------------------------------------**

***Recode Variables for Analysis**

***-------------------------------------------------**

**recode LowValueServiceCount (0=0 "No Low Value") (1/4=1 "At Least 1 Low Value"), gen(LowValueServiceRecode)**

**recode HighValueServiceCount (0=0 "No High Value") (1/max=1 "At Least 1 High Value"), gen(HighValueServiceRecode)**

**recode TOTCHRON (0=0 "No Chronic Conditions") (1=1 "1 Chronic Condition") (2/12 = 2 "2 or more Chronic Conditions") (-9=.), gen(TOTCHRONRECODE)**

**recode SEX (2=0 "Male") (1=1 "Female"), gen(FEMALE)**

**gen Eighteento64Pop2012 = .**

**replace Eighteento64Pop2012 = Eighteento34Pop2012 + ThirtyFiveto64Pop2012**

**gen Eighteento64Pop2013 = .**

**replace Eighteento64Pop2013 = Eighteento34Pop2013 + ThirtyFiveto64Pop2013**

**gen Eighteento64Pop2014 = .**

**replace Eighteento64Pop2014 = Eighteento34Pop2014 + ThirtyFiveto64Pop2014**

**gen Eighteento64Pop2015 = .**

**replace Eighteento64Pop2015 = Eighteento34Pop2015 + ThirtyFiveto64Pop2015**

**gen AdultPop2012 = .**

**replace AdultPop2012 = Eighteento34Pop2012 + ThirtyFiveto64Pop2012 + Over65Pop2012**

**gen AdultPop2013 = .**

**replace AdultPop2013 = Eighteento34Pop2013 + ThirtyFiveto64Pop2013 + Over65Pop2013**

**gen AdultPop2014 = .**

**replace AdultPop2014 = Eighteento34Pop2014 + ThirtyFiveto64Pop2014 +Over65Pop2014**

**gen AdultPop2015 = .**

**replace AdultPop2015 = Eighteento34Pop2015 + ThirtyFiveto64Pop2015 + Over65Pop2015**

**format AdultPop2012 AdultPop2013 AdultPop2013 AdultPop2015 %14.0f**

**gen AdultPop = .**

**replace AdultPop = AdultPop2012 if year == 2012**

**replace AdultPop = AdultPop2013 if year == 2013**

**replace AdultPop = AdultPop2014 if year == 2014**

**replace AdultPop = AdultPop2015 if year == 2015**

***Generate Visit Level Population Rate Variable**

**gen AdultPopSum = .**

**replace AdultPopSum = 1/55593582 if expansion2014 == 0 & year == 2012**

**replace AdultPopSum = 1/85089238 if expansion2014 == 1 & year == 2012**

**replace AdultPopSum = 1/56391500 if expansion2014 == 0 & year == 2013**

**replace AdultPopSum = 1/85830435 if expansion2014 == 1 & year == 2013**

**replace AdultPopSum = 1/57290748 if expansion2014 == 0 & year == 2014**

**replace AdultPopSum = 1/86555089 if expansion2014 == 1 & year == 2014**

**replace AdultPopSum = 1/58257821 if expansion2014 == 0 & year == 2015**

**replace AdultPopSum = 1/87200692 if expansion2014 == 1 & year == 2015**

***-------------------------------------------------**

***Save As Data Management File**

***-------------------------------------------------**
